# Temporal Matrix Stiffening Programs Osteogenic Differentiation and Immunomodulation to Enhance Osteogenesis

**DOI:** 10.3390/gels12100920

**Published:** 2026-10-14

**Authors:** Bofeng Pan, Jose-Enrique Rodarte, Jay Ming Tong, Ethan Nabeta, Dake Hao

**Affiliations:** 1 Department of Surgery, University of California Davis, Sacramento, CA 95817, USA; bfpan@health.ucdavis.edu (B.P.); josrodarte@ucdavis.edu (J.-E.R.); jaytong@ucdavis.edu (J.M.T.); enabeta@health.ucdavis.edu (E.N.); 2 Department of Biomedical Engineering, University of California Davis, Davis, CA 95616, USA; 3 Shriners Children’s, Northern California, Sacramento, CA 95817, USA

**Keywords:** temporal stiffening hydrogel, osteogenic differentiation, immunomodulation, BMSC, macrophage-to-BMSC paracrine signaling, osteogenesis

## Abstract

Dynamic changes in matrix mechanics play critical roles in bone regeneration, yet most biomaterial platforms provide static mechanical environments that fail to reflect the temporal evolution of the native healing niche. Here, we developed a dynamically stiffening GelMA-based hydrogel to investigate how temporally programmed matrix mechanics regulate bone marrow mesenchymal stem cell (BMSC) osteogenic differentiation and macrophage-to-BMSC paracrine signaling. The hydrogel initially provided a soft, permissive microenvironment that supported cell viability and spreading. Following an induced inflammatory challenge, the static-soft and dynamic conditions maintained macrophage phenotype-associated profiles more consistent with inflammation resolution than the static-stiff condition. Compared with continuous static stiffness, delayed matrix stiffening preserved greater macrophage and BMSC viability, maintained more favorable macrophage-derived paracrine signaling, and enhanced overall osteogenic outcomes. Both stiffened conditions promoted YAP nuclear localization, supporting the involvement of YAP-associated mechanotransduction. Collectively, these findings demonstrate that temporal mechanical programming can coordinate cellular adaptation, macrophage-associated inflammatory responses, and osteogenic differentiation, providing a promising design strategy for immunomodulatory biomaterials in bone tissue engineering and regenerative medicine.

## 1. Introduction

Critical-sized bone defects caused by trauma, tumor resection, infection, congenital malformation, or failed fracture healing remain among the most challenging problems in orthopedic and craniofacial reconstruction because they exceed the intrinsic regenerative capacity of bone and rarely heal without intervention [1]. In these settings, successful repair requires more than restoration of structural continuity. Bone regeneration must proceed through a tightly orchestrated sequence of events that includes control of early inflammation, recruitment of osteogenic progenitor cells, vascular invasion, extracellular matrix (ECM) deposition, and progressive tissue maturation under mechanical load [2]. Failure to coordinate these events frequently results in delayed union, non-union, fibrotic repair, or incomplete functional recovery, particularly in large segmental defects [3].

Autologous bone grafting remains the clinical benchmark for reconstruction because it uniquely combines osteogenic cells, osteoinductive factors, and an osteoconductive matrix within a single graft source [4]. However, its clinical use is limited by donor-site morbidity, restricted graft volume, increased operative time, and variable graft quality associated with patient age, comorbidity, and harvest location [5]. Allografts avoid the morbidity of autologous harvest and are widely used for structural reconstruction, but graft processing and sterilization reduce biological activity and may compromise remodeling, revascularization, incorporation, and long-term integration with host bone [6]. Synthetic substitutes based on ceramics, polymers, or composite scaffolds can provide structural support and design flexibility, yet most function primarily as space fillers or passive osteoconductive templates and do not recapitulate the dynamic biological and physicomechanical environment required to guide sequential phases of regeneration [7]. These limitations have driven growing interest in biomaterials that can actively instruct bone healing rather than merely occupy a defect [8].

Hydrogel scaffolds have emerged as attractive candidates for this purpose because they mimic several key features of native extracellular matrix, including high water content, three-dimensional architecture, and permeability to nutrients and soluble signals [9,10,11]. Their chemical versatility enables encapsulation of cells and bioactive factors while allowing control over adhesion, degradation, and mechanics in a form amenable to minimally invasive delivery and in situ gelation [12,13]. Among available hydrogel systems, gelatin methacryloyl (GelMA) is particularly well suited for bone-regenerative applications because it preserves gelatin-derived cell-adhesive motifs and enzymatically relevant sequences while introducing photocrosslinkable methacryloyl groups that permit rapid, controllable network formation [14]. As a result, GelMA can support cell attachment and remodeling while also enabling precise mechanical tuning through changes in polymer concentration, photoinitiator content, light intensity, and exposure time [15]. This combination of bioactivity and phototunability makes GelMA a rational platform for investigating how matrix mechanics regulate osteogenesis and osteoimmune signaling in three-dimensional culture. However, despite growing interest in biomaterial-mediated mechanoregulation, many studies examining stem cell responses to matrix mechanics have relied on conventional two-dimensional (2D) culture systems, which oversimplify the native cellular niche and do not fully recapitulate the three-dimensional architecture, matrix confinement, and cell–matrix interactions of ECM in vivo [16,17]. As a result, conclusions drawn from 2D systems may not fully translate to the complex mechanical and biochemical context of bone repair, where cells cultured in a three-dimensional (3D) matrix and experience spatially distributed cues from the surrounding microenvironment [18,19].

Bone-marrow-derived mesenchymal stem cells (BMSCs) provide a biologically relevant model for interrogating scaffold-mediated bone regeneration because they are clinically relevant osteogenic progenitors and are highly responsive to matrix mechanics [20]. Matrix stiffness regulates BMSC morphology, adhesion, cytoskeletal organization, proliferation, and lineage specification, and seminal mechanobiology studies have shown that cell fate can be directed by substrate elasticity [21]. These responses are mediated through integrin engagement, focal-adhesion signaling, actomyosin tension, and mechanosensitive transcriptional regulators such as yes-associated protein (YAP) and transcriptional coactivator with PDZ-binding motif (TAZ), which connect extracellular mechanical cues to osteogenic transcriptional programs [22,23]. These findings indicate that scaffold mechanics are not merely structural parameters, but active regulatory cues for BMSC osteogenic differentiation. A central challenge in scaffold design for MSC-mediated bone regeneration is that bone healing is not mechanically static [24]. During natural repair, the local microenvironment transitions from an initially compliant hematoma and provisional matrix toward a progressively stiffer callus and, ultimately, mineralized tissue capable of load bearing [25]. By contrast, most hydrogel scaffolds used to study or promote osteogenesis maintain a fixed mechanical state throughout culture or implantation [26]. This creates a fundamental design conflict. A compliant matrix can better support early cell viability, spreading, proliferation, and nutrient transport, thereby facilitating initial adaptation to a three-dimensional environment [27]. However, a persistently soft matrix may provide insufficient mechanical stimulation for robust osteogenic maturation [28]. Conversely, a stiff matrix can enhance osteogenic mechanotransduction and support matrix deposition and mineralization, but continuous exposure to a high-stiffness environment from the outset may constrain early spreading, reduce permissiveness for remodeling, and compromise the initial accommodation of encapsulated cells within a 3D environment [29,30]. Thus, the mechanical properties most favorable for late osteogenesis may be suboptimal during the earliest stages of repair. At the same time, bone regeneration is not governed by progenitor-cell mechanotransduction in isolation. The regenerative outcome is strongly shaped by osteoimmune interactions, particularly those involving macrophages, which participate in inflammatory signaling, debris clearance, angiogenesis, matrix remodeling, and the regulation of osteogenesis through paracrine communication with BMSCs [31]. M1-like macrophages contribute to early defense and inflammatory initiation, but prolonged inflammatory activation can impair osteogenesis and delay repair [32]. By contrast, M2-like macrophages are broadly associated with inflammation resolution, tissue remodeling, angiogenic support, and secretion of factors that favor BMSCs osteogenic differentiation [33]. Importantly, macrophages are also mechanosensitive. Changes in matrix mechanics can influence macrophage morphology, activation state, and secretory output, meaning that scaffold stiffness may regulate osteogenesis not only directly through BMSC mechanotransduction but also indirectly by reshaping the osteoimmune microenvironment [34,35]. This dual sensitivity makes the fixed-mechanics limitation of conventional scaffolds even more consequential: a stiff matrix may support osteogenic signaling yet simultaneously suppress pro-regenerative macrophage behavior, whereas a soft matrix may favor an immunologically supportive niche but underdeliver osteoinductive mechanical cues [36,37,38]. These observations point to a key knowledge gap in current biomaterial design. Mechanotransduction and osteoimmunology are both recognized as major regulators of bone repair, yet most scaffold systems do not integrate them in a temporally coordinated dynamical system [39,40,41]. A dynamically stiffening hydrogel offers a conceptually appealing solution because it can provide an early compliant microenvironment that supports cell spreading, viability, and pro-regenerative immune behavior, followed by a later increase in stiffness that promotes osteogenic mechanotransduction and matrix maturation [42]. This temporal-control framework is particularly well suited to dissect causal relationships between matrix mechanics, macrophage phenotype, and MSC osteogenesis.

**Figure 1:**
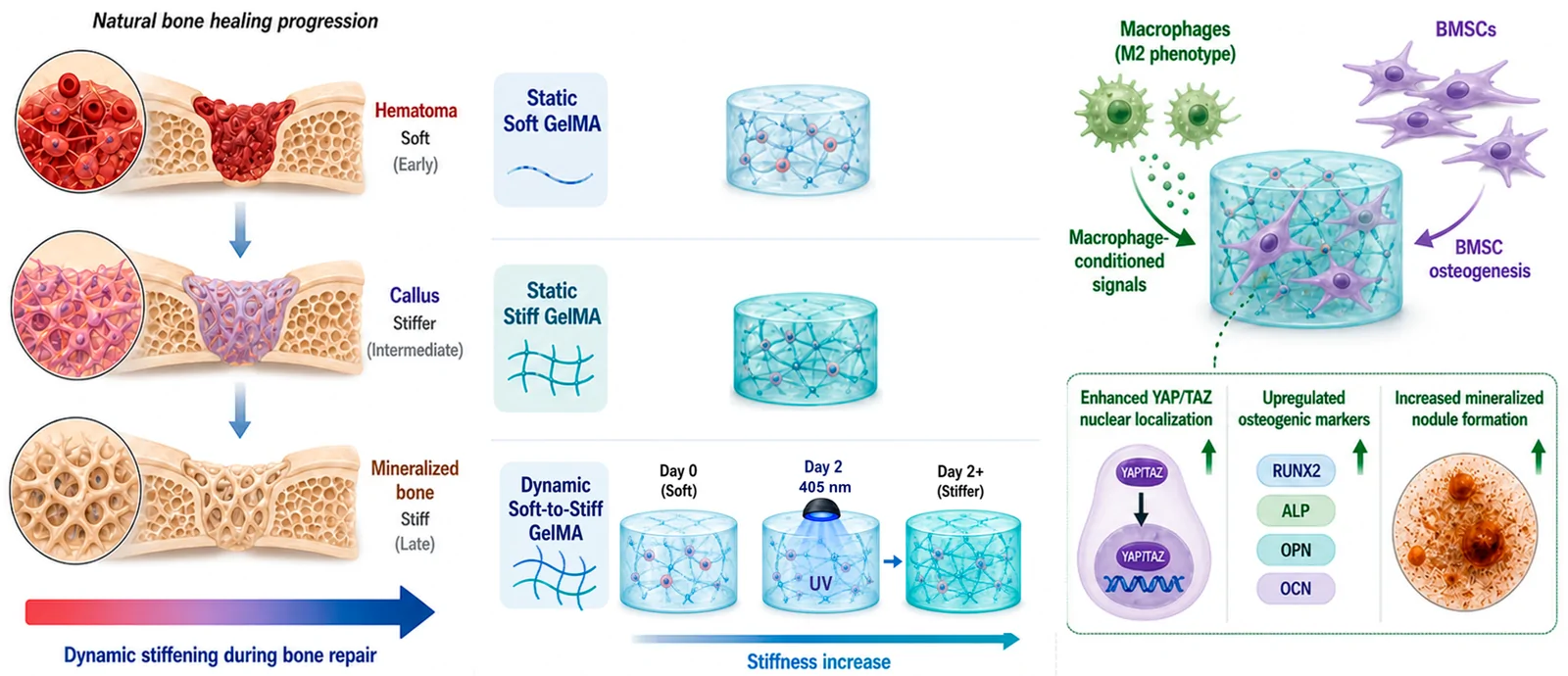
Schematic illustration of dynamic GelMA stiffening for temporal mechano-immune regulation of osteogenesis. Natural bone healing is accompanied by a progressive mechanical transition from an initially soft hematoma and provisional matrix to a stiffer callus and, ultimately, mineralized bone. Inspired by this temporal progression, three GelMA hydrogel conditions were designed: static-soft, static-stiff, and dynamic soft-to-stiff. Dynamic hydrogels were initially formed under the soft condition on Day 0 and subjected to secondary photocrosslinking with 405-nm light on Day 2 to increase matrix stiffness while maintaining the same material composition. The initial compliant phase was designed to support early BMSC adaptation and favorable macrophage responses following an inflammatory challenge, whereas delayed stiffening provided stronger mechanical stimulation associated with F-actin organization, YAP nuclear localization, ALP activity, OCN secretion, and matrix mineralization. The schematic presents dynamic matrix stiffening as a simplified, mechanically inspired strategy for coordinating macrophage-to-BMSC paracrine signaling with BMSC osteogenic differentiation.

Here, we developed a dynamically stiffening GelMA hydrogel platform to investigate the temporal regulation of BMSC osteogenesis and macrophage-to-BMSC paracrine signaling. Static-soft, static-stiff, and dynamic soft-to-stiff conditions were generated by varying the 405-nm photocrosslinking dose while maintaining a constant material composition, and hydrogel mechanics were quantified using oscillatory rheology (Figure 1). The dynamic group was initially formed under the soft condition and subsequently stiffened on Day 2. Using BMSC monoculture, THP-1-derived macrophages subjected to an LPS/IFN-γ-induced inflammatory challenge, and macrophage-conditioned-medium transfer to BMSCs, we tested whether temporal stiffening could preserve the early cytocompatibility and macrophage-associated advantages of a compliant matrix while subsequently providing mechanical stimulation that enhances osteogenic differentiation. YAP nuclear localization and pharmacological inhibition with Verteporfin were further evaluated to examine the contribution of YAP–TEAD-associated mechanotransduction to the observed osteogenic response [43]. By considering osteogenesis as the combined outcome of time-dependent mechanical cues and macrophage-derived signals, this study provides a design principle for developing next-generation immunomodulatory biomaterials for bone regeneration.

## 2. Results and Discussion

### 2.1. Preparation and Characterization of GelMA Hydrogels with Tunable Stiffness

**Figure 2:**
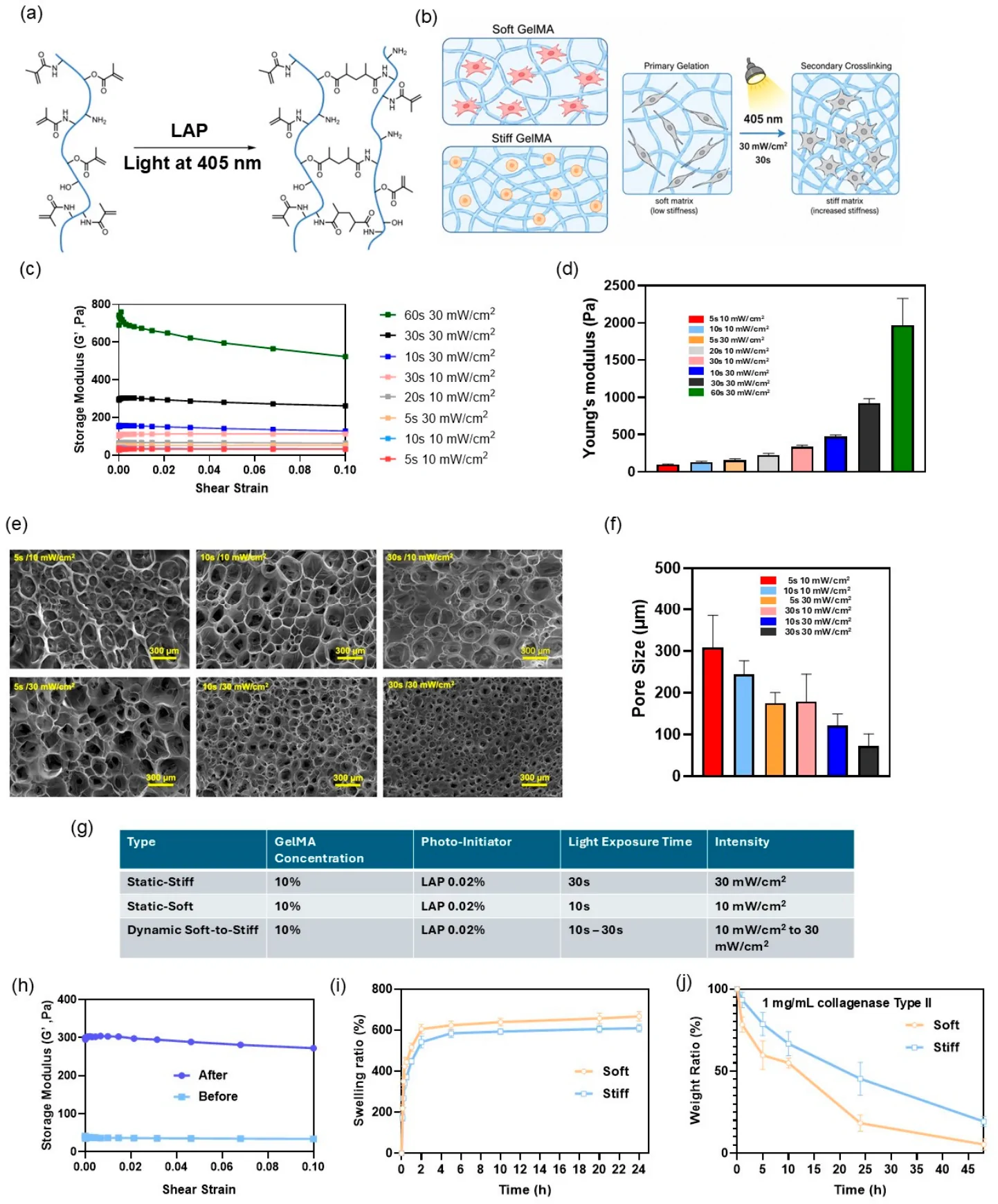
Preparation and physicochemical characterization of GelMA hydrogels with tunable stiffness. (**a**) Schematic illustration of LAP-mediated photocrosslinking of methacrylated gelatin (GelMA) under 405 nm light. (**b**) Schematic representation of static-soft and static-stiff GelMA networks and the dynamic soft-to-stiff condition. Dynamic stiffening was achieved by exposing a preformed soft hydrogel to secondary 405 nm irradiation at 30 mW/cm^2^ for 30 s. (**c**) Storage modulus (G’) of GelMA hydrogels prepared using different combinations of light intensity and exposure duration. (**d**) Young’s modulus estimated from the corresponding storage modulus values. (**e**) Representative scanning electron microscopy images showing the internal porous structures of GelMA hydrogels photocrosslinked for 5, 10, or 30 s at either 10 or 30 mW/cm^2^. Scale bars: 300 µm. (**f**) Quantification of hydrogel pore size under the indicated photocrosslinking conditions. (**g**) Summary of the GelMA formulation and photocrosslinking parameters used to generate the static-soft, static-stiff, and dynamic soft-to-stiff groups. All hydrogels contained 10% GelMA and 0.02% LAP. (**h**) Storage modulus of the dynamic hydrogels before and after secondary photocrosslinking. (**i**) Swelling kinetics of the static-soft and static-stiff hydrogels in PBS at 37 °C over 24 h. Swelling ratio was calculated relative to the initial lyophilized dry mass. (**j**) Enzymatic degradation profiles of the static-soft and static-stiff hydrogels in PBS containing 1 mg/mL collagenase Type II at 37 °C, expressed as the percentage of initial dry mass remaining. Data are presented as mean ± SD (*n* = 3 independent hydrogels per condition).

GelMA contains pendant methacryloyl groups that provide carbon–carbon double bonds capable of free-radical polymerization. Upon exposure to 405-nm light, LAP generates reactive radicals that initiate polymerization of these groups and form covalent crosslinks between neighboring GelMA chains, resulting in a three-dimensional hydrogel network (Figure 2a). Because the extent of crosslinking depends on the delivered light dose, hydrogel mechanics were tuned by varying light intensity and exposure duration while maintaining constant GelMA and LAP concentrations of 10% and 0.02% (*w*/*v*), respectively. This approach minimized compositional differences among the groups.

Selected photocrosslinking conditions were screened to identify mechanically distinct but experimentally suitable soft and stiff matrices (Figure 2b). Hydrogels were exposed to 405-nm light using combinations of 10 or 30 mW/cm^2^ and exposure durations ranging from 5 to 60 s. Rheological analysis demonstrated a light-dose-dependent increase in hydrogel stiffness (Figure 2c). Increasing either irradiation intensity or exposure duration increased the storage modulus (G′), with exposure at 30 mW/cm^2^ for 60 s producing the highest modulus and exposure at 10 mW/cm^2^ for 5 s producing the lowest modulus.

The apparent Young’s modulus estimated from G′ showed the same dose-dependent trend (Figure 2d). At 10 mW/cm^2^, increasing the exposure duration progressively increased the modulus from a highly compliant state to an intermediate mechanical range. Increasing the light intensity to 30 mW/cm^2^ produced a more pronounced increase, particularly at longer exposure durations. The 30 mW/cm^2^ for 60 s condition reached an apparent Young’s modulus of approximately 2 kPa and generated the stiffest network tested. However, this condition was not selected for cell studies because it produced a densely crosslinked microstructure, substantially reduced apparent pore size, and qualitatively increased brittleness during handling.

Scanning electron microscopy further demonstrated that the photocrosslinking dose regulated the apparent microarchitecture of the lyophilized hydrogels (Figure 2e). All formulations exhibited interconnected porous structures, whereas the average pore size decreased with increasing light intensity or exposure duration. At the lowest crosslinking dose, the hydrogels displayed relatively large, open pores with an average diameter of approximately 300 µm. Increasing the exposure duration at 10 mW/cm^2^ progressively reduced the apparent pore size. A more pronounced reduction was observed at 30 mW/cm^2^, for which the 30 s condition produced pores smaller than approximately 100 µm (Figure 2f). These results demonstrate that increasing the photocrosslinking dose increased hydrogel stiffness while decreasing the apparent pore size of the lyophilized structure. Because SEM was performed after freezing and lyophilization, these measurements do not directly represent the mesh size of the hydrated hydrogel network.

Based on the combined rheological, structural, and handling characteristics, exposure at 10 mW/cm^2^ for 10 s was selected for the static-soft group. Although the 5 s condition produced a lower modulus, the resulting hydrogels were less stable during handling. The 10 s condition generated a compliant but sufficiently stable network for three-dimensional culture and routine medium exchange. Exposure at 30 mW/cm^2^ for 30 s was selected for the static-stiff group because it produced a substantial increase in modulus while retaining an interconnected porous structure and avoiding the excessive densification and brittleness observed after 60 s. The selected static-soft and static-stiff hydrogels exhibited apparent Young’s moduli of 0.12 kPa and 0.9 kPa, respectively.

The selected conditions were subsequently used to establish the dynamic soft-to-stiff group (Figure 2b,g). Dynamic hydrogels were initially crosslinked at 10 mW/cm^2^ for 10 s, providing the same initial mechanical condition as the static-soft group. On Day 2, the preformed hydrogels underwent secondary photocrosslinking at 30 mW/cm^2^ for 30 s. Rheological measurements performed before and after secondary photocrosslinking confirmed that G′ increased from around 36 Pa to 300 Pa (Young’s modulus: 0.11 kPa to 0.9 kPa), corresponding to values comparable to those of the static-soft and static-stiff groups, respectively (Figure 2h). Thus, secondary photocrosslinking produced a substantial increase in matrix stiffness without altering the GelMA or LAP concentration. The experimental design therefore comprised a persistently compliant static-soft group, a persistently stiff static-stiff group, and a dynamic group that transitioned from a soft to a stiff mechanical state.

Hydrogel swelling and enzymatic degradation were subsequently evaluated to determine how the selected static-soft and static-stiff crosslinking conditions affected hydration and structural stability. Both groups rapidly absorbed PBS during the initial hours of incubation and approached equilibrium swelling within approximately 6 h (Figure 2i). The static-soft hydrogels exhibited a consistently higher swelling ratio than the static-stiff hydrogels, reaching an equilibrium swelling ratio of approximately 650%, compared with approximately 600% for the static-stiff condition. The lower swelling of the static-stiff hydrogels was consistent with their greater crosslink density and reduced capacity for network expansion.

Enzymatic degradation was evaluated in 1 mg/mL collagenase type II by measuring the remaining dry mass over 48 h (Figure 2j). Both groups underwent progressive mass loss, but the static-soft hydrogels degraded more rapidly than the static-stiff hydrogels. After 24 h, the static-soft hydrogels retained approximately 20% of their initial dry mass, whereas the static-stiff hydrogels retained approximately 40%. By 48 h, only a small fraction of the static-soft hydrogel mass remained, whereas the static-stiff hydrogels retained approximately 15%. These findings demonstrate that increasing the photocrosslinking dose reduced hydrogel swelling and increased resistance to collagenase-mediated degradation.

The differences in apparent pore structure, swelling, and degradation indicate that photocrosslinking altered multiple physical properties in addition to stiffness. These changes may influence cellular confinement, matrix remodeling, and the transport of nutrients and soluble factors. Although increasing the photocrosslinking dose reduced the apparent pore size, the static-stiff hydrogels retained an interconnected porous structure with pores on the order of 100 µm. These pores are substantially larger than typical nutrients and soluble proteins and are therefore expected to remain permissive to molecular transport through the water-rich GelMA network. However, SEM measurements of lyophilized samples do not directly represent the mesh size or diffusion properties of hydrated hydrogels, and oxygen and nutrient transport were not measured directly. Therefore, the observed biological responses cannot be attributed exclusively to stiffness, and the potential contributions of accompanying changes in network architecture and transport properties should also be considered.

Collectively, the rheological, microstructural, swelling, and degradation results confirmed that the selected photocrosslinking conditions generated GelMA hydrogels with distinct physical properties. The static-soft condition provided a compliant, highly hydrated, and more rapidly degradable matrix, whereas the static-stiff condition provided greater mechanical reinforcement and enzymatic stability. Secondary photocrosslinking enabled a substantial temporal increase in matrix stiffness, supporting the use of the dynamic soft-to-stiff condition in subsequent macrophage and BMSC experiments.

### 2.2. Matrix Mechanics Regulate Macrophage Viability and Polarization

**Figure 3:**
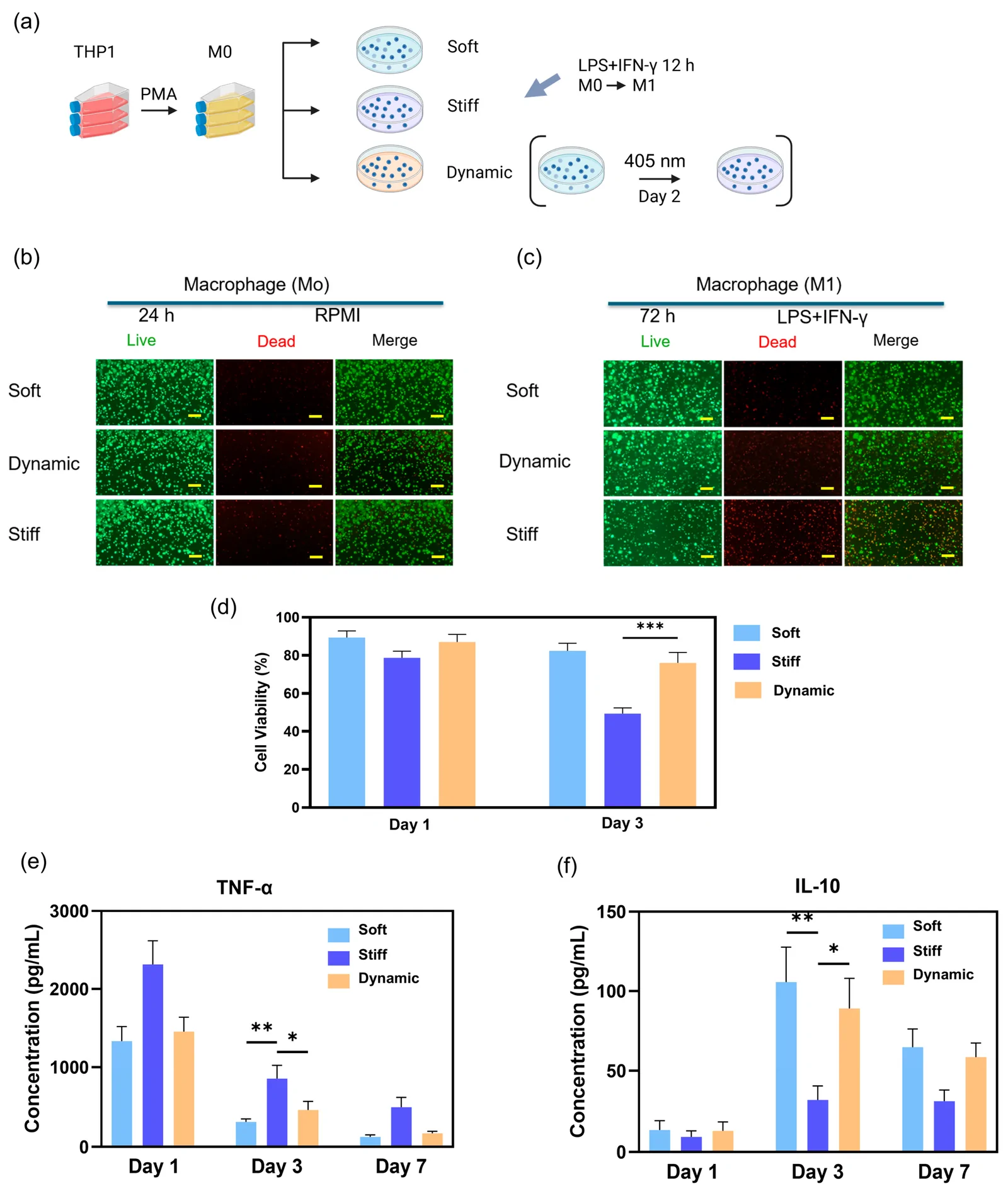
Matrix stiffness regulates macrophage viability and inflammatory cytokine secretion. (**a**) Schematic of the macrophage culture. THP-1 monocytes were differentiated into M0-like macrophages using phorbol 12-myristate 13-acetate (PMA), detached, and encapsulated within static-soft, static-stiff, or dynamic soft-to-stiff GelMA hydrogels. The dynamic group underwent secondary photocrosslinking on Day 2 using 405 nm light at 30 mW/cm^2^ for 30 s. M1-like activation was induced by treatment with lipopolysaccharide (LPS) and interferon-γ (IFN-γ) for 12 h. (**b**) Representative Live/Dead fluorescence images of M0-like macrophages after 24 h of culture in RPMI-1640 medium under the indicated GelMA conditions. Scale bar: 200 µm (**c**) Representative Live/Dead images of M1-like macrophages after 72 h of culture. Viable and dead cells are shown in green and red, respectively. (**d**) Quantification of macrophage viability on Days 1 and 3. (**e**,**f**) TNF-α and IL-10 concentrations, respectively, in macrophage culture supernatants collected on Days 1, 3, and 7. Data are presented as mean ± SD (*n* = 3). Statistical significance was determined using two-way ANOVA followed by Tukey’s multiple-comparisons test. * *p* < 0.05, ** *p* < 0.01, and *** *p* < 0.001.

The effects of static and temporally changing matrix stiffness on macrophage behavior were evaluated using M0-like macrophages differentiated from THP-1 monocytes and subsequently encapsulated within static-soft, static-stiff, or dynamic soft-to-stiff GelMA hydrogels (Figure 3a). For the dynamic group, secondary photocrosslinking was performed on Day 2. The encapsulated macrophages were challenged with LPS and IFN-γ for 12 h to induce an M1-like inflammatory response. Thus, this model was used to assess how matrix mechanics influenced macrophage responses during and after a standardized inflammatory challenge, rather than spontaneous polarization in the absence of inflammatory stimulation. Live/Dead staining showed that macrophages remained predominantly viable under all three hydrogel conditions after 24 h of culture, with relatively few dead cells (Figure 3b). On Day 1, cell viability was approximately 89%, 78%, and 87% in the static-soft, static-stiff, and dynamic groups, respectively (Figure 3d). Although viability tended to be lower in the static-stiff group, no significant differences were detected among the groups at this early time point.

Following inflammatory activation, macrophage viability remained relatively high in the static-soft and dynamic groups at 72 h, whereas substantially more dead cells were observed in the static-stiff group (Figure 3c). On Day 3, viability was approximately 82% in the static-soft group and 76% in the dynamic group but decreased to approximately 49% in the static-stiff group. Macrophage viability in the static-stiff group was significantly lower than that in the dynamic group (Figure 3d). These results suggest that sustained exposure to the static-stiff matrix compromised macrophage survival, whereas delayed stiffening preserved a greater proportion of the cytocompatibility associated with the initial soft environment.

To determine whether the 405-nm light exposure itself (higher dose) contributed to the observed viability differences, macrophages (M0) were either exposed to 405-nm light at 30 mW/cm^2^ for 30 s on Day 2 or maintained without light exposure. Live/Dead analysis performed 24 h later showed no significant difference in cell viability between the light-exposed and no-light groups (Figure S1b). Therefore, under the conditions used in this study, the light exposure did not produce a detectable reduction in macrophage viability. This control supports the interpretation that the lower viability observed in the static-stiff group was primarily associated with sustained matrix conditions rather than acute cytotoxicity caused by the 405-nm irradiation protocol.

Matrix mechanics also influenced cytokine secretion following LPS/IFN-γ stimulation. TNF-α was evaluated as a pro-inflammatory cytokine associated with M1-like activation, whereas IL-10 was evaluated as an anti-inflammatory and inflammation-resolving cytokine. TNF-α concentrations were highest on Day 1 in all groups, consistent with the initial inflammatory challenge, and subsequently decreased on Days 3 and 7 (Figure 3e). However, macrophages maintained within the static-stiff matrix exhibited the highest TNF-α concentration, particularly on Day 3, when TNF-α levels were significantly higher than those in the static-soft and dynamic groups. These findings indicate that continuous exposure to the static-stiff environment was associated with a more persistent pro-inflammatory response.

In contrast, IL-10 secretion increased after the initial inflammatory phase and reached its highest level on Day 3 (Figure 3f). The static-soft group exhibited the highest IL-10 concentration, followed by the dynamic group, whereas the static-stiff group showed substantially lower IL-10 secretion. On Day 3, IL-10 concentrations in both the static-soft and dynamic groups were significantly higher than those in the static-stiff group. This cytokine profile suggests that the initially compliant environments more effectively supported the development of an inflammation-resolving response following the LPS/IFN-γ challenge.

Immunofluorescence analysis of macrophage phenotype-associated markers on Day 5 further supported the cytokine results (Figure S2a). Expression of the M1-associated costimulatory molecule CD86 was highest in the static-stiff group and significantly greater than that in both the static-soft and dynamic groups (Figure S2b). In contrast, expression of the M2-associated scavenger receptor CD163 was lowest in the static-stiff group, whereas the static-soft and dynamic groups exhibited significantly higher CD163 fluorescence intensities (Figure S2c). No significant difference was observed between the static-soft and dynamic groups for either marker. Together, the increased CD86 and reduced CD163 expression in the static-stiff group were consistent with maintenance of a more pro-inflammatory phenotype, whereas the dynamic group retained a marker profile more similar to that of the static-soft group.

The cytokine and immunofluorescence findings should be interpreted together with the observed differences in macrophage viability. Because secreted cytokines were measured as concentrations in the culture supernatant rather than normalized to viable cell number, the lower viability of the static-stiff group may have contributed to its reduced IL-10 concentration. Nevertheless, the static-stiff group exhibited the highest TNF-α concentration despite containing fewer viable cells, together with increased CD86 and decreased CD163 expression. These convergent findings support an association between sustained matrix stiffness and a predominantly pro-inflammatory macrophage response that cannot be explained solely by differences in cell survival.

The distinct macrophage responses may reflect the combined effects of matrix mechanics and microstructure. The static-stiff hydrogels exhibited a higher modulus, lower swelling, slower degradation, and smaller apparent pore size than the static-soft hydrogels. These properties may have imposed greater mechanical confinement and restricted cellular accommodation within the three-dimensional network. In contrast, the softer and more hydrated matrix provided a more permissive initial environment that preserved macrophage viability and supported an inflammation-resolving secretory and marker profile.

Importantly, the dynamic soft-to-stiff group exhibited macrophage responses more similar to those of the static-soft group than to those of the static-stiff group. Although secondary photocrosslinking increased matrix stiffness on Day 2, the dynamic group maintained relatively high viability, lower TNF-α secretion, higher IL-10 secretion, lower CD86 expression, and higher CD163 expression compared with the static-stiff group. These findings suggest that the initial period of matrix compliance influenced the subsequent macrophage response and that delayed stiffening did not reproduce the adverse effects associated with continuous exposure to a stiff matrix.

This distinction was relevant to the subsequent macrophage-conditioned-medium experiments. Prolonged pro-inflammatory macrophage signaling may impair BMSC function, whereas an inflammation-resolving secretory profile may be more supportive of tissue repair and osteogenic differentiation. The dynamic condition therefore provided a temporal combination of an initially permissive environment for macrophages and subsequent mechanical reinforcement for BMSCs. Overall, the results indicate that temporal stiffening preserved a macrophage phenotype-associated profile established during the initial soft phase while avoiding the sustained inflammatory response observed under static-stiff conditions.

### 2.3. Effects of Static and Dynamic Matrix Stiffness on BMSC Viability, Spreading, and Osteogenic Differentiation

**Figure 4:**
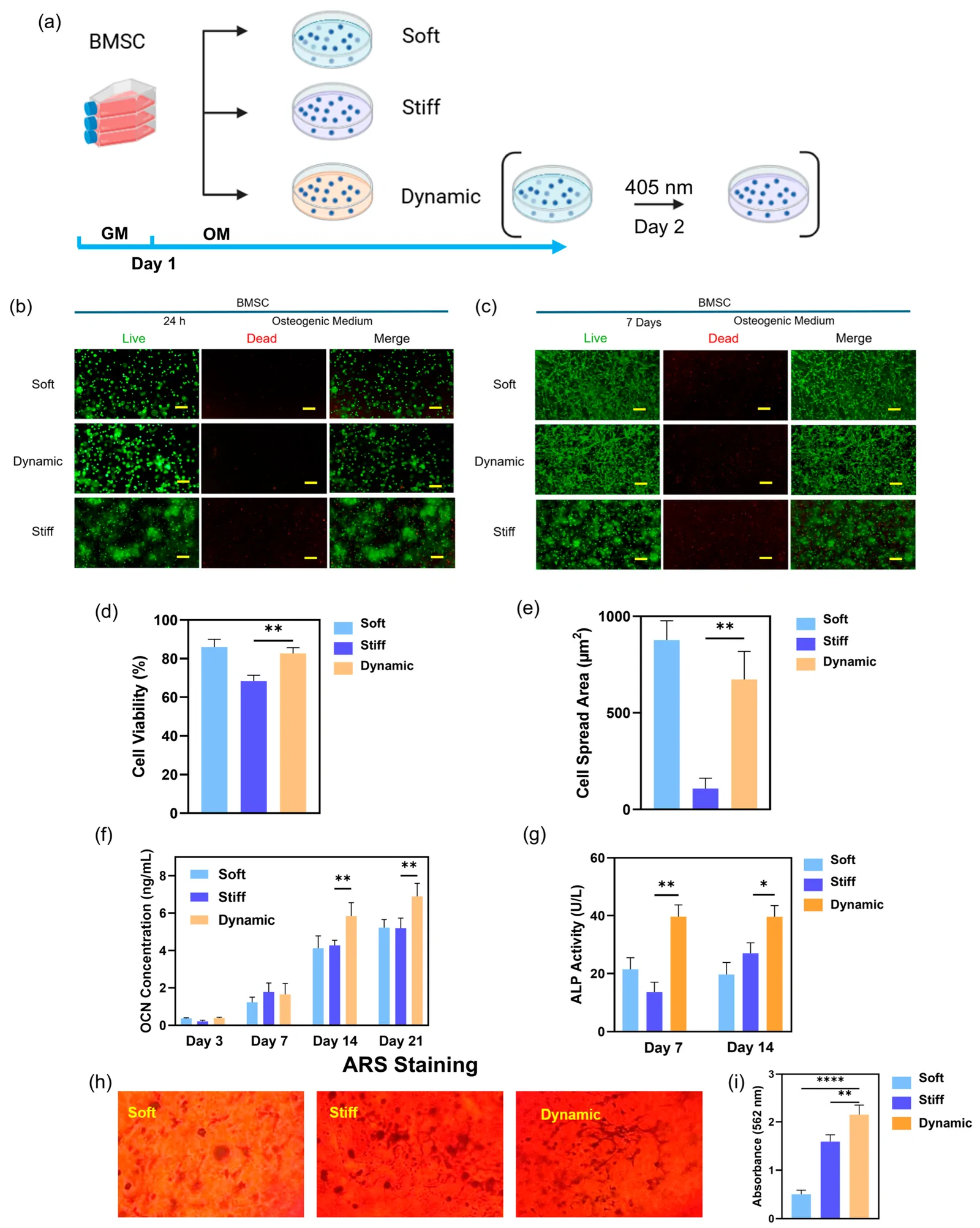
Dynamic matrix stiffening preserves BMSC viability and spreading while promoting osteogenic differentiation. (**a**) Schematic illustration of BMSC culture within static-soft, static-stiff, and dynamic soft-to-stiff GelMA hydrogels. Cell-laden hydrogels were initially maintained in growth medium (GM) and switched to osteogenic medium (OM) on Day 1. The dynamic group underwent secondary photocrosslinking on Day 2 to increase matrix stiffness. (**b**,**c**) Representative Live/Dead fluorescence images of BMSCs after 24 h and 7 days of culture, respectively, under the indicated GelMA conditions. Viable cells are shown in green and dead cells in red. Scale bar: 200 μm. (**d**) Quantification of BMSC viability on Day 7. (**e**) Quantification of projected BMSC spreading area after 7 days of culture. (**f**) Osteocalcin (OCN) concentrations measured on Days 3, 7, 14, and 21. (**g**) Alkaline phosphatase (ALP) activity measured on Days 7 and 14. (**h**) Representative Alizarin Red S staining images showing matrix mineralization under static-soft, static-stiff, and dynamic soft-to-stiff conditions. (**i**) Quantification of Alizarin Red S staining following dye extraction with cetylpyridinium chloride (CPC) and measurement of absorbance at 562 nm. Data are presented as mean ± SD (*n* = 3). Panels (**d**,**e**,**i**) were analyzed using one-way ANOVA followed by Tukey’s multiple-comparisons test. Panels (**f**,**g**) were analyzed using two-way ANOVA followed by Tukey’s multiple-comparisons test. * *p* < 0.05, ** *p* < 0.01, and **** *p* < 0.0001.

The effects of static and temporally changing matrix stiffness on BMSC viability, morphology, and osteogenic differentiation were evaluated using the experimental design shown in Figure 4a. BMSCs were encapsulated within static-soft, static-stiff, or dynamic soft-to-stiff GelMA hydrogels, initially maintained in growth medium, and switched to osteogenic medium on Day 1. The dynamic group underwent secondary photocrosslinking on Day 2, thereby increasing matrix stiffness after an initial period of culture within the compliant hydrogel.

Live/Dead staining showed that BMSCs remained predominantly viable under all three hydrogel conditions after 24 h of culture, although more dead cells were visible in the static-stiff group than in the static-soft and dynamic groups (Figure 4b). After 7 days of osteogenic culture, extensive calcein-positive fluorescence was retained in the static-soft and dynamic groups, whereas the static-stiff group exhibited a greater proportion of dead cells and less extensive cell spreading (Figure 4c).

Quantitative analysis confirmed that BMSC viability on Day 7 was highest in the static-soft group, followed by the dynamic group, and lowest in the static-stiff group (Figure 4d). Cell viability was approximately 86% in the static-soft group and 82% in the dynamic group but decreased to approximately 68% in the static-stiff group. Viability in the dynamic group was significantly higher than that in the static-stiff group (*p* < 0.01), indicating that delayed matrix stiffening was better tolerated than continuous exposure to the static-stiff environment.

To evaluate whether the light exposure itself affected BMSC viability, matched BMSC were either exposed to 405-nm light at 30 mW/cm^2^ for 30 s on Day 2 or maintained without light exposure. Live/Dead analysis performed 24 h later showed no significant difference in cell viability between the light-exposed and no-light groups (Figure S1a). Therefore, the light irradiation protocol did not produce a detectable reduction in BMSC viability under the conditions used in this study. This control supports the interpretation that the differences among the hydrogel groups were primarily associated with their matrix conditions rather than acute cytotoxicity caused by 405-nm light exposure.

Matrix mechanics also strongly influenced BMSC morphology. Cells cultured in static-soft hydrogels displayed the largest projected spreading area, whereas BMSCs in static-stiff hydrogels remained comparatively rounded and exhibited limited spreading (Figure 4e). The dynamic group retained substantial cell spreading after secondary stiffening and exhibited a significantly greater projected cell area than the static-stiff group. These findings suggest that the initial soft phase provided a permissive environment for cellular accommodation and morphological development and that much of this early spreading advantage was retained after delayed stiffening.

The influence of matrix mechanics on osteogenic differentiation was subsequently evaluated by measuring osteocalcin secretion (OCN), alkaline phosphatase (ALP) activity, and matrix mineralization. OCN concentrations increased progressively from Day 3 to Day 21 in all groups, indicating time-dependent osteogenic maturation (Figure 4f). At the early time points, OCN concentrations were relatively low and similar among the groups. By Days 14 and 21, however, the dynamic group exhibited the highest OCN concentrations, whereas the static-soft and static-stiff groups showed lower and broadly comparable values. This temporal pattern suggests that delayed stiffening enhanced late-stage osteogenic maturation after an initial period of culture within a compliant matrix.

ALP activity also varied according to the mechanical condition (Figure 4g). On Day 7, the dynamic group exhibited the highest ALP activity, followed by the static-soft group, whereas the static-stiff group showed the lowest activity. By Day 14, ALP activity decreased in the static-soft group but increased in the static-stiff and dynamic groups. The dynamic group maintained the highest mean ALP activity at both time points, consistent with an enhanced early-to-intermediate osteogenic response under dynamic stiffening.

Alizarin Red S staining on Day 21 demonstrated mineral deposition under all three matrix conditions (Figure 4h). More extensive staining was visually observed in the static-stiff and dynamic groups than in the static-soft group. To quantitatively assess mineralization, the bound Alizarin Red S was extracted using cetylpyridinium chloride (CPC) and measured at 562 nm (Figure 4i). The dynamic group exhibited the highest absorbance, followed by the static-stiff and static-soft groups. Mineralization in the dynamic group was significantly greater than that in both the static-soft and static-stiff groups, confirming that delayed matrix stiffening produced the strongest mineralization response.

Collectively, these findings demonstrate that continuous exposure to a static-stiff matrix reduced BMSC viability and restricted cell spreading, despite supporting greater mineral deposition than the static-soft condition. In contrast, the static-soft matrix favored early viability and spreading but provided weaker mechanical stimulation for later-stage mineralization. The dynamic condition combined these advantages by providing an initially compliant environment that supported early cellular adaptation, followed by increased matrix stiffness that promoted ALP activity, OCN secretion, and mineral deposition. The absence of a detectable viability effect from the 405-nm exposure control further indicates that these outcomes were associated with temporal changes in matrix mechanics rather than light-induced cytotoxicity. Thus, temporal stiffening more effectively coordinated early BMSC survival and spreading with subsequent osteogenic differentiation than either static mechanical condition alone.

### 2.4. Dynamic Matrix Stiffening Enhances Macrophage-to-BMSC Paracrine Support of Osteogenic Differentiation

**Figure 5:**
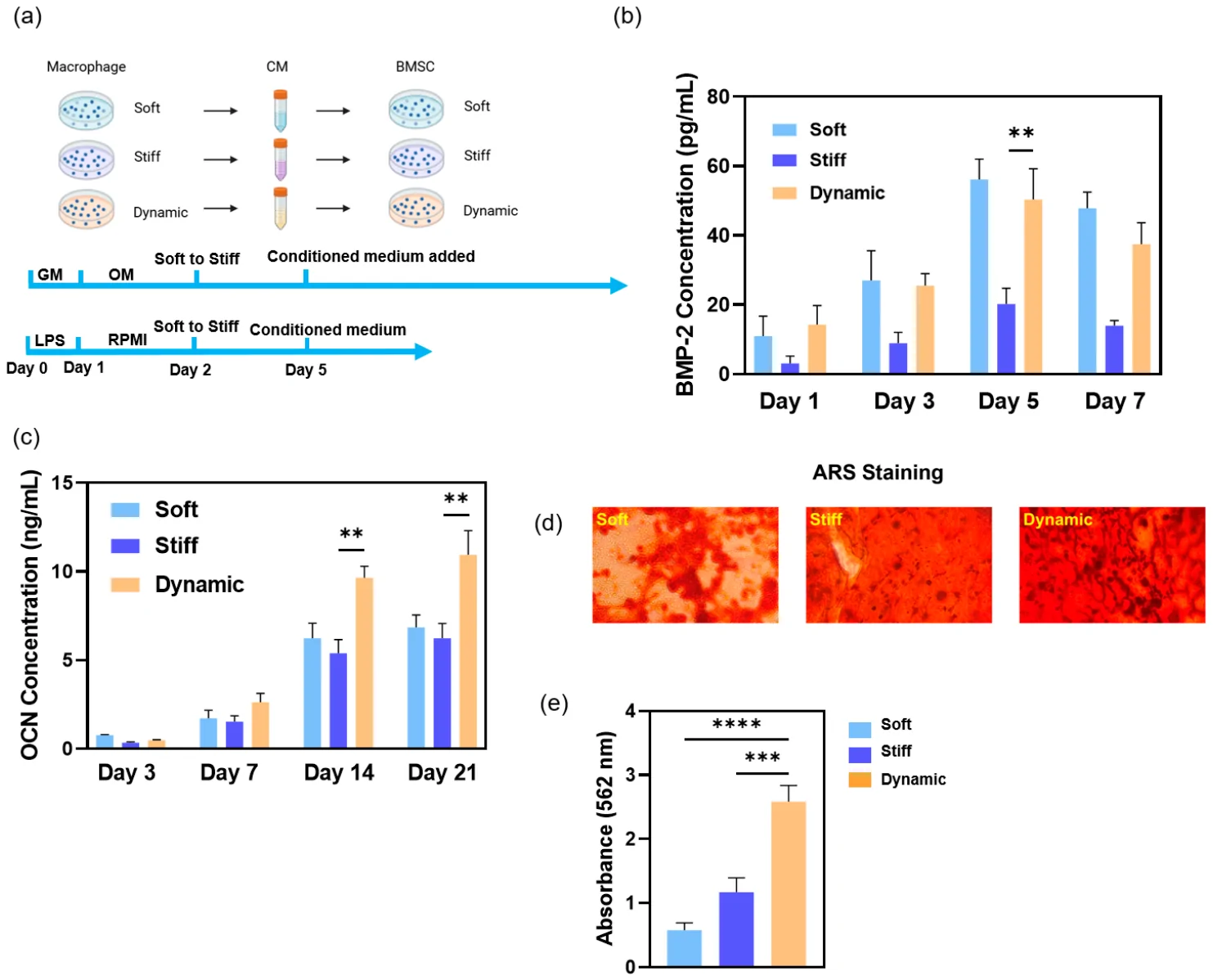
Dynamic matrix stiffening enhances macrophage-conditioned-medium-mediated BMSC osteogenic differentiation. (**a**) Schematic illustration of the macrophage-conditioned-medium model. Macrophages were cultured within static-soft, static-stiff, or dynamic soft-to-stiff GelMA hydrogels. Conditioned medium (CM) was collected on Day 5 and mixed at a 1:1 ratio with fresh osteogenic medium before being applied to BMSCs cultured under the corresponding hydrogel condition. In both macrophage and BMSC cultures, the dynamic group underwent secondary photocrosslinking on Day 2 using 405 nm light at 30 mW/cm^2^ for 30 s. (**b**) BMP-2 concentrations in macrophage culture supernatants collected on Days 1, 3, 5, and 7. (**c**) Osteocalcin (OCN) concentrations in BMSC culture supernatants measured on Days 3, 7, 14, and 21. (**d**) Representative Alizarin Red S staining images showing mineral-associated calcium deposition after 21 days of osteogenic culture. (**e**) Quantification of Alizarin Red S staining following dye extraction with CPC and measurement of absorbance at 562 nm. Data are presented as mean ± SD (*n* = 3). Panels (**b**,**c**) were analyzed using two-way ANOVA with hydrogel condition and time as factors, followed by Tukey’s multiple-comparisons test. Panel (**e**) was analyzed using one-way ANOVA followed by Tukey’s multiple-comparisons test. ** *p* < 0.01, *** *p* < 0.001, and **** *p* < 0.0001.

To investigate macrophage-to-BMSC paracrine signaling under different mechanical conditions, macrophages and BMSCs were cultured separately within matched static-soft, static-stiff, or dynamic soft-to-stiff GelMA hydrogels (Figure 5a). In both cell models, the dynamic groups underwent secondary stiffening on Day 2. Macrophage-conditioned medium was collected on Day 5, mixed at a 1:1 ratio with fresh osteogenic medium, and applied to BMSCs cultured under the corresponding matched hydrogel condition.

BMP-2 secretion by macrophages differed among the three mechanical environments during conditioned-medium generation (Figure 5b). The static-soft and dynamic groups consistently exhibited higher BMP-2 concentrations than the static-stiff group. In the static-soft and dynamic groups, BMP-2 concentrations increased from Day 1 to Day 5 and declined moderately by Day 7. The static-soft group reached the highest mean BMP-2 concentration on Day 5, whereas the dynamic group maintained comparatively elevated BMP-2 secretion from Days 3 to 7. In contrast, BMP-2 secretion remained relatively low in the static-stiff group throughout the observation period. At the Day 5 conditioned-medium collection time point, BMP-2 concentration in the dynamic group was significantly higher than that in the static-stiff group.

The increased BMP-2 secretion observed in the static-soft and dynamic groups was consistent with their macrophage cytokine and phenotype-associated marker profiles. Under these conditions, macrophages exhibited lower TNF-α secretion, higher IL-10 secretion, lower CD86 expression, and higher CD163 expression than those maintained in the static-stiff hydrogels. These findings suggest that the initially compliant matrix supported an inflammation-resolving, pro-regenerative macrophage response accompanied by greater production of osteoinductive soluble factors. However, because conditioned medium contains numerous cytokines and growth factors, and BMP-2 activity was not selectively inhibited, the present results identify BMP-2 as a plausible contributor rather than establishing it as the sole mediator of the subsequent BMSC response.

OCN secretion by BMSCs increased over the culture period under all three matched mechanical conditions (Figure 5c). The Day 3 measurement preceded the addition of macrophage-conditioned medium on Day 5 and therefore represents an early baseline osteogenic response. On Day 7, after approximately 2 days of conditioned-medium exposure, OCN concentrations remained relatively low, although the dynamic group exhibited a higher mean value than the static groups. By Days 14 and 21, OCN secretion in the dynamic group was significantly higher than that in the static-stiff group. These results indicate that the dynamic condition produced the strongest late-stage osteogenic response following macrophage-conditioned-medium treatment.

Alizarin Red S staining on Day 21 further demonstrated matrix mineralization under all three conditions (Figure 5d). The dynamic group showed the most extensive and intense staining, consistent with enhanced deposition of calcium-rich mineral. To quantify this response, bound Alizarin Red S was extracted using CPC and measured at 562 nm (Figure 5e). The dynamic group exhibited the highest absorbance, followed by the static-stiff and static-soft groups. Mineralization in the dynamic group was significantly greater than that in both the static-soft and static-stiff groups, confirming that temporal stiffening enhanced late-stage mineral deposition in the macrophage-conditioned-medium model.

The enhanced osteogenic response under dynamic stiffening likely reflected the combined influence of macrophage-derived soluble factors and direct mechanical regulation of BMSCs. The static-soft condition preserved macrophage and BMSC viability, supported greater cell spreading, and promoted relatively high macrophage BMP-2 secretion. However, persistent matrix compliance may have provided insufficient mechanical resistance to maximize BMSC mechanotransduction and mineralization. Conversely, the static-stiff condition provided stronger mechanical stimulation but reduced macrophage and BMSC viability, restricted BMSC spreading, and was associated with increased TNF-α, increased CD86, decreased IL-10 and CD163, and reduced BMP-2 secretion.

The dynamic soft-to-stiff condition balanced these competing effects. During the initial soft phase, macrophages and BMSCs maintained relatively high viability and adapted to the three-dimensional environment. By the Day 5 conditioned-medium collection point, macrophages in the dynamic group retained a more inflammation-resolving and osteoinductive secretory profile than those in the static-stiff group. Secondary stiffening simultaneously provided stronger mechanical stimulation to BMSCs during subsequent osteogenic maturation. The combination of these effects was associated with increased OCN secretion and the greatest mineral deposition.

Because conditioned medium from each macrophage group was transferred only to BMSCs cultured under the corresponding matched hydrogel condition, this experiment evaluated the combined effects of macrophage-derived paracrine signaling and BMSC matrix mechanics. It did not independently separate the contribution of conditioned-medium composition from the direct effect of the recipient hydrogel environment. A cross-transfer design in which each conditioned medium is applied across all three BMSC matrix conditions, together with selective inhibition of candidate mediators such as BMP-2, would be required to establish their individual contributions. Nevertheless, the present findings demonstrate that temporal matrix stiffening generated a coordinated macrophage–BMSC microenvironment associated with stronger osteogenic maturation than either static-soft or static-stiff conditions.

### 2.5. YAP-Associated Mechanotransduction Contributes to Dynamic-Stiffening-Enhanced Osteogenic Differentiation

**Figure 6:**
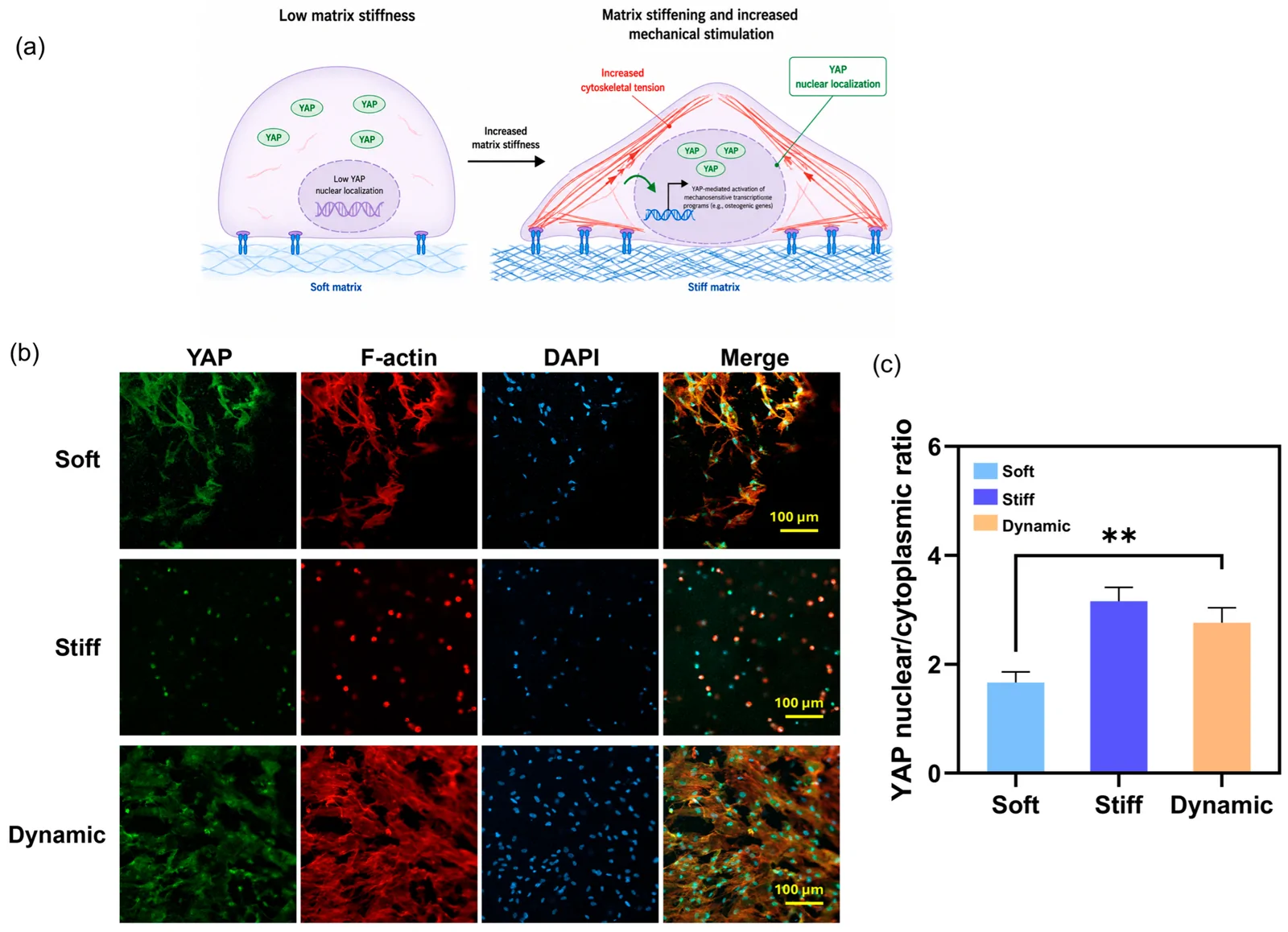
Dynamic matrix stiffening promotes YAP nuclear localization and F-actin organization in BMSCs exposed to macrophage-conditioned medium. (**a**) Schematic illustration of stiffness-dependent YAP mechanotransduction. Under low matrix stiffness, reduced cytoskeletal tension is associated with limited YAP nuclear localization. Matrix stiffening increases cytoskeletal tension and promotes YAP translocation into the nucleus, where YAP activates mechanosensitive transcriptional programs associated with osteogenic differentiation. (**b**) Representative immunofluorescence images of YAP (green), F-actin (red), and nuclei stained with DAPI (blue) in BMSCs cultured under static-soft, static-stiff, or dynamic soft-to-stiff GelMA conditions in the presence of matched macrophage-conditioned medium. Merged images show the spatial relationship among YAP, the actin cytoskeleton, and nuclei. (**c**) Quantification of the YAP nuclear-to-cytoplasmic fluorescence intensity ratio. Data are presented as mean ± SD (*n* = 3); measurements from multiple imaging fields were averaged to obtain one value for each hydrogel. Statistical significance was determined using one-way ANOVA followed by Tukey’s multiple-comparisons test. ** *p* < 0.01.

To investigate the mechanism underlying the enhanced osteogenic response observed in the macrophage-conditioned-medium model, YAP localization and F-actin organization were examined in BMSCs cultured within static-soft, static-stiff, and dynamic soft-to-stiff GelMA hydrogels in the presence of matched macrophage-conditioned medium. The schematic in Figure 6a illustrates the proposed mechanotransduction mechanism, in which increased matrix stiffness enhances cytoskeletal tension and promotes YAP nuclear translocation, thereby activating mechanosensitive transcriptional programs associated with osteogenic differentiation.

Immunofluorescence staining revealed distinct differences in YAP localization and actin cytoskeletal organization among the three mechanical conditions (Figure 6b). BMSCs cultured in static-soft hydrogels exhibited relatively diffuse YAP fluorescence, with a substantial proportion of the signal remaining in the cytoplasm. These cells also displayed comparatively less organized F-actin, consistent with the lower resistance to forces provided by the compliant matrix. In contrast, BMSCs cultured in static-stiff hydrogels exhibited greater nuclear accumulation of YAP, indicating enhanced stiffness-sensitive mechanotransduction. However, F-actin organization and cell spreading remained limited relative to the dynamic group, consistent with the reduced viability and projected spreading area observed under continuous exposure to the static-stiff matrix.

The dynamic soft-to-stiff group exhibited extensive F-actin organization together with strong nuclear enrichment of YAP. Quantitative analysis showed that the YAP nuclear-to-cytoplasmic fluorescence intensity ratio was approximately 1.7 in the static-soft group, compared with approximately 3.2 and 2.9 in the static-stiff and dynamic groups, respectively (Figure 6c). Thus, the static-stiff group exhibited the highest mean ratio, while the dynamic group also showed substantially greater YAP nuclear localization than the static-soft group. The difference between the dynamic and static-soft groups was statistically significant. These results demonstrate that both continuous stiffness and delayed stiffening promoted YAP nuclear localization, whereas the dynamic condition combined strong YAP activation with more extensive F-actin organization and better preservation of cell viability and spreading.

To examine the functional contribution of YAP–TEAD signaling to osteogenic differentiation, BMSCs cultured under each matched mechanical and conditioned-medium condition were treated with 0.5 µM Verteporfin (Figure S3a). For the dynamic group, secondary photocrosslinking was completed before Verteporfin was added, preventing exposure of the compound to the 405-nm light used for matrix stiffening. Verteporfin significantly reduced ALP activity in the static-stiff and dynamic groups, with the greatest reduction observed under the dynamic condition, whereas the decrease in the static-soft group was not statistically significant (Figure S3b). A similar pattern was observed for OCN secretion: Verteporfin significantly reduced OCN concentrations in the static-stiff and dynamic groups but did not produce a significant change in the static-soft group (Figure S3c). These findings indicate that the enhanced osteogenic responses associated with matrix stiffening were particularly sensitive to pharmacological inhibition of YAP–TEAD signaling.

The Verteporfin results complement the YAP localization data and support a functional contribution of YAP-associated mechanotransduction to osteogenic differentiation. During the initial soft phase, BMSCs were able to survive, spread, and establish cell–matrix interactions before exposure to stronger mechanical stimulation. Subsequent stiffening increased matrix resistance to cell-generated traction, promoted actin cytoskeletal organization, and enhanced YAP nuclear localization. Nuclear YAP can interact with TEAD transcription factors to regulate mechanosensitive programs involved in cytoskeletal remodeling, cell–matrix adhesion, and osteogenic differentiation. The marked reduction in ALP activity and OCN secretion following Verteporfin treatment suggests that YAP–TEAD transcriptional activity contributed to the osteogenic advantage of the dynamic condition.

The macrophage-conditioned-medium results further suggest that this mechanical response occurred within a biochemical environment shaped by macrophage-derived soluble factors. Macrophages cultured under the dynamic condition maintained greater viability and a less pro-inflammatory phenotype-associated profile than those continuously exposed to the static-stiff matrix while also producing comparatively high levels of BMP-2. The conditioned-medium and osteogenic-medium mixture may therefore have supplied biochemical signals that cooperated with stiffness-induced mechanotransduction in BMSCs. BMP-2 represents one plausible contributor through SMAD-dependent osteogenic signaling, whereas matrix stiffening promoted F-actin organization and YAP nuclear localization. Because BMP-2 activity was not selectively inhibited, however, its interaction with YAP signaling remains a proposed mechanism rather than a directly established causal relationship.

Notably, the static-stiff group exhibited the highest mean YAP nuclear-to-cytoplasmic ratio but did not achieve the strongest overall osteogenic outcome. This finding indicates that YAP nuclear localization alone was insufficient to determine the final response. Continuous exposure to the static-stiff matrix enhanced mechanical signaling but simultaneously reduced BMSC viability and spreading and generated a less favorable macrophage-derived secretory environment. In contrast, the dynamic condition produced slightly lower YAP nuclear localization than the static-stiff condition but combined substantial YAP activation with greater cell viability, more extensive F-actin organization, and more favorable macrophage-derived paracrine signaling. This combination was associated with higher ALP activity, OCN secretion, and mineral deposition.

Although Verteporfin is widely used as a pharmacological inhibitor of YAP–TEAD signaling, it may also exert YAP-independent effects. Therefore, the inhibition results provide supportive pharmacological evidence rather than definitive proof that all observed effects were mediated exclusively by YAP. Future studies using genetic YAP/TAZ silencing and measurements of canonical YAP–TEAD target genes would help confirm pathway specificity.

Taken together, these findings support a coordinated mechanism in which temporal matrix stiffening promotes F-actin organization and YAP-associated mechanotransduction in BMSCs while preserving a macrophage-derived paracrine environment favorable to osteogenesis. The dynamic matrix therefore enhanced osteogenic differentiation through the integration of temporal mechanical stimulation and macrophage-derived biochemical signals.

## 3. Conclusions

In this study, we developed a GelMA hydrogel platform that enabled temporal control of matrix stiffness while maintaining constant GelMA and LAP concentrations. By varying the 405-nm photocrosslinking dose, we generated mechanically distinct static-soft and static-stiff matrices, while delayed secondary photocrosslinking produced a dynamic soft-to-stiff transition. Rheological, microstructural, swelling, and degradation analyses confirmed that the selected photocrosslinking conditions generated hydrogels with distinct mechanical and physical properties. This platform provided a controlled model for investigating how the timing of mechanical stimulation regulates macrophage responses and BMSC osteogenic differentiation.

The static-soft matrix supported cell viability and spreading and was associated with a more inflammation-resolving macrophage profile, characterized by lower TNF-α and CD86 and higher IL-10, CD163, and BMP-2 levels than the static-stiff condition. In contrast, continuous exposure to the static-stiff matrix reduced macrophage and BMSC viability, restricted BMSC spreading, and maintained a more pro-inflammatory macrophage response. These findings indicate that increased matrix stiffness alone is insufficient to optimize bone-regenerative responses and may be detrimental when strong mechanical constraints are imposed during early cellular adaptation.

The dynamic soft-to-stiff condition provided a more favorable balance between early cytocompatibility and subsequent mechanical stimulation. The initial compliant phase preserved macrophage and BMSC viability, supported BMSC spreading, and maintained a macrophage-derived secretory environment more favorable to osteogenesis than that produced under continuous static stiffness. Subsequent stiffening enhanced ALP activity, OCN secretion, and quantitatively measured mineral deposition. In the matched macrophage-conditioned-medium model, the dynamic condition produced the strongest overall osteogenic response, supporting the combined importance of macrophage-derived paracrine signals and BMSC matrix mechanics.

Mechanistically, delayed stiffening promoted F-actin organization and YAP nuclear localization in BMSCs. Pharmacological inhibition with Verteporfin substantially reduced ALP activity and OCN secretion under the dynamic condition, supporting a functional contribution of YAP–TEAD-associated mechanotransduction to the enhanced osteogenic response. Nevertheless, the static-stiff group exhibited the highest mean YAP nuclear-to-cytoplasmic ratio without achieving the strongest osteogenic outcome, indicating that YAP nuclear localization alone was insufficient. Instead, the benefits of dynamic stiffening appeared to arise from the integration of mechanical stimulation with improved cell survival, cytoskeletal organization, and macrophage-derived paracrine support.

Overall, these findings identify the temporal sequence of matrix mechanics as an important determinant of macrophage and BMSC behavior. An initially soft and permissive environment followed by delayed mechanical reinforcement preserved early cytocompatibility and a favorable macrophage phenotype-associated profile while subsequently enhancing osteogenic mechanotransduction and mineralization. Dynamic matrix stiffening therefore represents a promising design strategy for bone-regenerative biomaterials intended to coordinate early cellular adaptation and inflammatory regulation with later-stage osteogenic maturation. Future studies should use cross-transfer conditioned-medium designs, genetic YAP/TAZ perturbation, selective inhibition of candidate paracrine mediators such as BMP-2, and in vivo bone-defect models to further define the underlying mechanisms and therapeutic potential.

## 4. Materials and Methods

### 4.1. Materials

PhotoGel^®^ methacrylated gelatin (GelMA; approximately 95% degree of methacrylation, sterile and lyophilized) was purchased from Advanced BioMatrix (Carlsbad, CA, USA). Lithium phenyl-2,4,6-trimethylbenzoylphosphinate (LAP), bovine serum albumin (BSA), RPMI-1640 medium containing L-glutamine and sodium bicarbonate, phorbol 12-myristate 13-acetate (PMA; P1585), recombinant human interferon-γ (IFN-γ; SRP3058), lipopolysaccharide from *Escherichia coli* O111 (LPS; L2630), Mesenchymal Stem Cell Osteogenic Differentiation Medium supplied as a ready-to-use kit containing basal medium and SupplementMix (C-28013), Alkaline Phosphatase Assay Kit (MAK447), Alizarin Red S solution (2003999), cetylpyridinium chloride monohydrate (CPC; C0732), collagenase from *Clostridium histolyticum*, Type II (C6885), and Verteporfin (SML0534) were purchased from MilliporeSigma (Burlington, MA, USA). The human THP-1 monocytic cell line (TIB-202), normal human bone-marrow-derived mesenchymal stem cells (BMSCs; PCS-500-012), Mesenchymal Stem Cell Basal Medium (PCS-500-030), and Mesenchymal Stem Cell Growth Kit for Bone Marrow-derived MSCs (PCS-500-041) were obtained from the American Type Culture Collection (ATCC, Manassas, VA, USA). DuoSet^®^ ELISA development kits for human tumor necrosis factor-α (TNF-α), interleukin-10 (IL-10), bone morphogenetic protein-2 (BMP-2), and osteocalcin (OCN) were purchased from R&D Systems (Minneapolis, MN, USA). The Alexa Fluor^®^ 488-conjugated YAP (D8H1X) rabbit monoclonal antibody (14729) was purchased from Cell Signaling Technology (Danvers, MA, USA). For macrophage phenotype-associated immunofluorescence staining, a phycoerythrin-conjugated mouse monoclonal antibody against human B7-2/CD86 (FAB141P) was purchased from R&D Systems. A rabbit monoclonal antibody against CD163 (clone EPR19518; ab182422) was purchased from Abcam (Cambridge, UK). The Alexa Fluor™ 488-conjugated goat anti-rabbit IgG (H + L) cross-adsorbed secondary antibody (A-11008) and ProLong™ Diamond Antifade Mountant with DAPI (P36962) were obtained from Thermo Fisher Scientific/Invitrogen (Waltham, MA, USA). Heat-inactivated fetal bovine serum, calcein-AM, propidium iodide, fluorescent phalloidin reagent, Triton™ X-100 Surfact-Amps™ detergent solution, and 4% paraformaldehyde in PBS were purchased from Thermo Fisher Scientific (Waltham, MA, USA).

### 4.2. Preparation and Photocrosslinking of GelMA Hydrogels

PhotoGel^®^ methacrylated gelatin was dissolved in sterile 1× PBS to prepare a 10% (*w*/*v*) GelMA precursor solution. Briefly, sterile PBS was prewarmed to above 60 °C and added to the lyophilized GelMA. The mixture was gently agitated on a rotator or shaker until the GelMA was completely dissolved and was maintained above 37 °C during preparation to prevent premature physical gelation.

LAP was dissolved separately in sterile 1× PBS and added to the GelMA solution to achieve a final concentration of 0.02% (*w*/*v*). The precursor solution was mixed until homogeneous and protected from light before use. GelMA and LAP concentrations were maintained constant across all groups, and hydrogel stiffness was controlled exclusively by varying the photocrosslinking intensity and exposure duration, thereby minimizing compositional differences among the experimental conditions.

The precursor solution was dispensed into 96 well plate at 70 µL per hydrogel and photocrosslinked using a portable 405 nm light source (EFL-LS-1601-405; ANR Technologies Pte Ltd., Singapore). Static-soft hydrogels were photocrosslinked at 10 mW/cm^2^ for 10 s, whereas static-stiff hydrogels were photocrosslinked at 30 mW/cm^2^ for 30 s. Dynamic soft-to-stiff hydrogels were initially photocrosslinked at 10 mW/cm^2^ for 10 s and subsequently subjected to secondary photocrosslinking on Day 2 at 30 mW/cm^2^ for 30 s.

The dynamic hydrogels therefore remained under the soft condition during the initial period and underwent delayed stiffening on Day 2 without changes in GelMA concentration, LAP concentration, or material formulation. Static-soft and static-stiff hydrogels were not subjected to secondary photocrosslinking, and all groups were otherwise handled under identical experimental conditions.

### 4.3. Rheological Characterization

The viscoelastic properties of the GelMA hydrogels were characterized using a rotational rheometer (MCR 92, Anton Paar, Graz, Austria) equipped with a 25 mm parallel-plate geometry. Hydrogels prepared using different combinations of light intensity and exposure duration were analyzed, including crosslinking times of 5, 10, 20, 30 and 60 s at light intensities of 10 or 30 mW/cm^2^. The hydrogel samples were placed between the rheometer plates, and the measuring gap was set to 1 mm.

Oscillatory strain-sweep measurements were performed over a strain range of 0.01–100% using 25 logarithmically distributed measurement points at a constant angular frequency of 10 rad/s. Storage modulus (G′) and loss modulus (G″) were recorded as functions of shear strain. The representative storage modulus for each hydrogel condition was determined from the plateau region within the linear viscoelastic range.

Young’s modulus was estimated from the storage modulus by assuming that the GelMA hydrogels behaved as isotropic, nearly incompressible elastic materials with a Poisson’s ratio of 0.5. Accordingly, Young’s modulus was calculated using:

*E* = 2*G*^′^(1 + *ν*)

where *E* is Young’s modulus, *G*^′^ is the storage modulus measured within the linear viscoelastic region, and *ν* is Poisson’s ratio. With *ν* = 0.5, the relationship simplifies to:

*E* = 3*G*^′^

At least 3 independently prepared hydrogel samples were analyzed for each condition, and the results are presented as mean ± SD.

### 4.4. Swelling Ratio Measurement

The swelling behavior of the GelMA hydrogels was evaluated using acellular samples. Hydrogels of 500 μL were prepared under the static-soft and static-stiff photocrosslinking conditions described above. Following photocrosslinking, the hydrogels were immersed in PBS at 37 °C for 24 h to remove soluble and unreacted components, with the PBS replaced once after 6 h. The samples were subsequently rinsed three times with deionized water for 5 min per wash to remove residual salts.

The washed hydrogels were frozen at −80 °C and lyophilized for 24 h until a constant dry mass was obtained. Each dried hydrogel was weighed using an analytical balance, and the initial dry mass was recorded as Wd.

Each dried hydrogel was then completely immersed in 2 mL of PBS and incubated at 37 °C. The swollen mass was measured after 0.5, 1, 2, 4, 6, 12, and 24 h. At each time point, the hydrogel was carefully removed from PBS, and excess surface liquid was removed by gently blotting each surface with lint-free tissue for 5 s without compressing the sample. The hydrogel was immediately weighed, and the swollen mass at each time point was recorded as W_s_(t). After weighing, the hydrogel was returned to the same container containing PBS.

The swelling ratio was calculated as follows:

Swelling ratio (%) = [(W_s_(t) − Wd)/Wd] × 100

where Wd represents the initial lyophilized dry mass and W_s_(t) represents the swollen mass measured at the indicated time point.

### 4.5. Enzymatic Degradation Assay

The enzymatic degradation of the GelMA hydrogels was evaluated using collagenase Type II. Acellular hydrogels of identical volume and geometry were prepared under the static-soft and static-stiff photocrosslinking conditions described above. Following photocrosslinking, the hydrogels were incubated in PBS at 37 °C for 24 h to remove soluble and unreacted components, with the PBS replaced once after approximately 6 h.

Because dry-mass determination is destructive, separate hydrogels were prepared for the baseline and each degradation time point. To determine the initial dry mass, matched time-zero hydrogels from each condition were rinsed three times with deionized water for 5 min per wash, frozen at −80 °C, and lyophilized for 24 h until a constant mass was obtained. The mean dry mass of the time-zero hydrogels in each group was recorded as W_0_.

A freshly prepared collagenase solution containing 1 mg/mL collagenase from *Clostridium histolyticum*, Type II (C6885; MilliporeSigma), was prepared in 1× PBS supplemented with 0.36 mM CaCl_2_. Each hydrogel was completely immersed in 200 μL of collagenase solution and incubated at 37 °C under gentle agitation. The collagenase solution was replaced with freshly prepared solution after 24 h.

Hydrogels were collected after 5, 10, 24, and 48 h of enzymatic degradation. At each time point, the remaining hydrogel and all visible fragments were carefully recovered. The samples were washed once with cold PBS and subsequently washed three times with deionized water for 5–10 min per wash to remove residual collagenase, calcium salts, and PBS components. The recovered samples were transferred into preweighed tubes, frozen at −80 °C, and lyophilized for 24 h until a constant mass was obtained. The remaining dry mass at each time point was recorded as W_t_.

The remaining weight ratio was calculated as follows:

Weight ratio (%) = (W_t_/W_0_) × 100

where W_0_ represents the mean initial dry mass of the matched time-zero hydrogels and W_t_ represents the remaining dry mass after collagenase treatment for the indicated duration.

### 4.6. Scanning Electron Microscopy and Pore-Size Analysis

GelMA hydrogels prepared under the indicated photocrosslinking conditions were rapidly frozen in liquid nitrogen and immediately transferred to a freeze-dryer for lyophilization for 24 h. The lyophilized samples were sectioned to expose their internal porous structures, mounted on aluminum stubs, sputter-coated with gold, and imaged using a Quattro S scanning electron microscope (Thermo Fisher Scientific, Waltham, MA, USA) at an accelerating voltage of 15 kV.

Representative SEM images were acquired under identical imaging conditions for all groups. Hydrogel pore size was quantified from the SEM images using ImageJ (ImageJ version 1.54p; National Institutes of Health, Bethesda, MD, USA). Images were calibrated using the embedded scale bars, and individual pores were manually outlined after threshold-based identification. Pore size was expressed as the equivalent circular diameter calculated from the measured pore area. For each independently prepared hydrogel, at least 50 pores from five randomly selected fields were analyzed. Three independently prepared hydrogels were evaluated per condition, and the mean pore size calculated for each hydrogel was used as the independent value for statistical analysis. Data are presented as mean ± SD.

### 4.7. Cell Culture, Differentiation, and Encapsulation in GelMA Hydrogels

Human THP-1 monocytes were obtained from ATCC and maintained in RPMI-1640 medium supplemented with 10% fetal bovine serum and 1% penicillin–streptomycin. Primary human bone-marrow-derived mesenchymal stem cells (BMSCs) were obtained from ATCC as well and cultured in MSC Basal Medium supplemented with the corresponding growth supplement and 0.1% penicillin–streptomycin. Both cell types were maintained at 37 °C in a humidified atmosphere containing 5% CO_2_. BMSCs between passages 2 and 4 were used for all experiments.

THP-1 monocytes were seeded in tissue-culture flasks and treated with phorbol 12-myristate 13-acetate (PMA) at 150 ng/mL for 24 h to induce differentiation into adherent M0-like macrophages. Following PMA treatment, the cells were washed with PBS and rested in PMA-free RPMI-1640 medium for 24 h. The differentiated M0-like macrophages were detached by incubation with Accutase for 30 min at 37 °C, collected by centrifugation, and resuspended in the GelMA/LAP precursor solution at a density of 2 × 10^6^ cells/mL. A 70 µL volume of the cell-containing precursor, corresponding to 1.4 × 10^5^ macrophages per hydrogel, was dispensed and photocrosslinked under the designated static-soft, static-stiff, or dynamic soft-to-stiff conditions. For M1-like activation, the encapsulated M0-like macrophages were subsequently stimulated with lipopolysaccharide (LPS) at 100 ng/mL and interferon-γ (IFN-γ) at 20 ng/mL for 12 h.

BMSCs were suspended in the GelMA/LAP precursor solution at a density of 1 × 10^5^ cells per 70 µL hydrogel and encapsulated by photocrosslinking under the indicated mechanical conditions. The cell-laden hydrogels were initially maintained in growth medium and switched on Day 1 to commercially available Mesenchymal Stem Cell Osteogenic Differentiation Medium containing basal medium and SupplementMix. Osteogenic medium was replaced every 3 days throughout the culture period.

For the dynamic soft-to-stiff condition, cell-laden hydrogels were initially photocrosslinked under the static-soft condition and subjected to secondary photocrosslinking on Day 2 at 30 mW/cm^2^ for 30 s. Culture medium was removed before secondary light exposure. Fresh medium was added immediately after photocrosslinking, and the cultures were returned to the incubator. Static-soft and static-stiff hydrogels were not subjected to secondary photocrosslinking and were otherwise handled in parallel.

### 4.8. Live/Dead Viability Assay

Cell viability was evaluated using calcein-AM and propidium iodide (PI) staining. A freshly prepared staining solution containing 2 µM calcein-AM and 2 µg/mL PI in PBS or phenol red-free medium was used for all experiments. Cell-laden hydrogels were gently washed once with PBS and incubated with the staining solution for 30 min at 37 °C while protected from light. After staining, the samples were gently washed three times with PBS and maintained in fresh PBS during imaging.

Fluorescence images were acquired immediately using a Zeiss fluorescence microscope. Calcein-positive viable cells were detected using the FITC/GFP channel with excitation at approximately 488 nm, whereas PI-positive dead cells were detected using the TRITC/Texas Red channel. Live cells were visualized by green cytoplasmic fluorescence, while dead cells were identified by red nuclear fluorescence. Macrophage viability was assessed on Days 1 and 3, and BMSC viability was evaluated after 24 h and on Day 7.

Cell viability was calculated as:

$$\mathrm{Cell viability}\left(\%\right)=\frac{N_{\mathrm{live}}}{N_{\mathrm{live}}+N_{\mathrm{dead}}}\times 100$$

For each sample, at least 10 randomly selected fields were analyzed using ImageJ/Fiji. Three independently prepared cell-laden hydrogels were evaluated per group, and the mean value calculated from the analyzed fields of each hydrogel was treated as one independent replicate. Data are presented as mean ± SD.

### 4.9. Quantification of BMSC Spreading Area

BMSC spreading was evaluated from fluorescence images acquired after 7 days of culture. Cell boundaries were identified from calcein-AM-positive cytoplasmic fluorescence in the Live/Dead images. Images were calibrated using the embedded scale bar, converted to grayscale, and processed using a consistent intensity threshold in ImageJ/Fiji. Individual cell outlines were then manually verified and adjusted when necessary to ensure accurate segmentation.

The projected spreading area of each cell was measured in square micrometers. Cells touching the image boundary, cells that could not be clearly distinguished from adjacent cells, and overlapping cell clusters that prevented reliable segmentation were excluded from the analysis. For each independently prepared hydrogel, at least 30 clearly identifiable cells from five randomly selected fields were analyzed. Three independently prepared hydrogels were evaluated per group, and the mean projected cell area calculated for each hydrogel was treated as one independent replicate. Data are presented as mean ± SD.

Because the BMSCs were encapsulated within three-dimensional GelMA hydrogels, the measurement is reported as projected cell area rather than total cell surface area or cell volume.

### 4.10. Collection and Application of Macrophage-Conditioned Medium

M0-like macrophages encapsulated within static-soft, static-stiff, or dynamic soft-to-stiff GelMA hydrogels were stimulated with LPS at 100 ng/mL and IFN-γ at 20 ng/mL for 12 h to induce an M1-like inflammatory response. Following stimulation, the hydrogels were washed three times with sterile PBS to remove residual LPS and IFN-γ and were subsequently incubated in fresh RPMI-1640 medium. For the dynamic group, secondary photocrosslinking was performed on Day 2 at 30 mW/cm^2^ for 30 s, as described above.

Macrophage-conditioned medium was collected on Day 5 after two days of incubation in fresh RPMI-1640 medium. The collected supernatants were centrifuged at 1500 rpm for 5 min to remove suspended cells and cellular debris. The clarified conditioned medium was either used immediately or aliquoted and stored at −80 °C until further analysis. Repeated freeze–thaw cycles were avoided.

For the macrophage-to-BMSC paracrine signaling experiment, conditioned medium obtained from macrophages cultured under each mechanical condition was applied to BMSCs maintained under the corresponding matched GelMA condition. Conditioned medium from the static-soft, static-stiff, and dynamic macrophage groups was therefore transferred to BMSCs cultured in static-soft, static-stiff, and dynamic hydrogels, respectively. On Day 5, the BMSC culture medium was replaced with a 1:1 mixture of macrophage-conditioned medium and fresh osteogenic differentiation medium. The conditioned-medium mixture was renewed every 3 days throughout the remaining culture period.

### 4.11. ELISA-Based Quantification of Secreted Cytokines and Osteogenic Factors

The concentrations of TNF-α, IL-10, BMP-2, and osteocalcin (OCN) in culture supernatants were quantified using commercially available DuoSet^®^ ELISA development kits (R&D Systems, Minneapolis, MN, USA). Samples were collected at the designated time points, centrifuged to remove suspended cells and debris, aliquoted, and stored at −80 °C until analysis. Repeated freeze–thaw cycles were avoided.

For macrophage cultures, TNF-α and IL-10 concentrations were measured in macrophage-conditioned medium collected on Days 1, 3, and 7. TNF-α was used as an indicator of pro-inflammatory cytokine secretion, whereas IL-10 was used as an indicator of anti-inflammatory or inflammation-resolving cytokine secretion. The results were interpreted as changes in macrophage inflammatory cytokine secretion rather than as definitive evidence of macrophage phenotype.

BMP-2 concentrations were measured on Days 1, 3, 5, and 7 in macrophage-conditioned medium. OCN secretion by BMSCs was measured in culture supernatants collected on Days 3, 7, 14, and 21 as an indicator of osteogenic differentiation.

Samples and standards were analyzed in triplicate. Absorbance was measured at 450 nm using a SpectraMax® i3 Multi-Mode Microplate Reader (Molecular Devices, LLC, San Jose, CA, USA). Analyte concentrations were calculated from standard curves. TNF-α, IL-10, and BMP-2 concentrations were reported in pg/mL, whereas osteocalcin concentrations were reported in ng/mL. Osteocalcin values were normalized by cell numbers.

### 4.12. Alkaline Phosphatase Activity

Alkaline phosphatase (ALP) activity was measured on Days 7 and 14 using a commercially available Alkaline Phosphatase Assay Kit (MAK447; MilliporeSigma, Burlington, MA, USA). Cell-laden GelMA hydrogels were washed with PBS and digested with collagenase type II at 37 °C to release the encapsulated BMSCs. The recovered cells were collected by centrifugation and lysed in 0.2% Triton X-100 in purified water by shaking for 20 min at room temperature. The lysates were subsequently clarified by centrifugation, and the supernatants were collected for analysis.

Samples and calibrators were assayed in duplicate in clear, flat-bottom 96-well plates. After addition of the assay working reagent containing assay buffer, magnesium acetate, and p-nitrophenyl phosphate, absorbance at 405 nm was measured immediately and again after 4 min using a microplate reader.

ALP activity was calculated according to the equation:

$$\mathrm{ALP activity}(U/L)=\frac{(OD_{T4}-OD_{T0})\times \mathrm{RxnVol}\times 35.3}{(OD_{\mathrm{Cal}}-OD_{\mathrm{Blank}})\times \mathrm{SmplVol}\times T}$$

where *OD_T_*_4_ and *OD_T_*_0_ represent the sample absorbance values at 4 and 0 min, respectively, *OD*_Cal_ and *OD*_Blank_ represent the calibrator and blank absorbance values, RxnVol is the final reaction volume, SmplVol is the sample volume, and *T* is the reaction time. ALP activity was reported in U/L.

### 4.13. Alizarin Red S Staining and CPC-Based Quantification

Matrix mineralization was evaluated by Alizarin Red S (ARS) staining on Day 21. BMSC-laden GelMA hydrogels were gently washed two to three times with PBS for 5 min per wash to remove residual culture medium and serum. The hydrogels were then fixed with 4% paraformaldehyde for 30 min at room temperature and washed three times with PBS.

The fixed hydrogels were completely immersed in 2% ARS solution adjusted to pH 4.1–4.3 and incubated for 60 min at room temperature with gentle rocking. Excess dye was removed by repeatedly washing the hydrogels with deionized water until the wash solution became nearly clear. The hydrogels were subsequently washed overnight in deionized water at 4 °C, with the water replaced twice to further reduce nonspecific background.

Following the final wash, the stained hydrogels were imaged under identical illumination, magnification, and acquisition settings. For quantitative analysis, the bound ARS dye was extracted using 10% (*w*/*v*) cetylpyridinium chloride (CPC) prepared in 10 mM sodium phosphate buffer at pH 7.0. Each hydrogel was transferred into an individual container and completely immersed in 500 µL of CPC extraction solution. Samples were incubated for 1 h at room temperature with gentle agitation to extract the bound ARS dye.

After extraction, the solution was mixed thoroughly, and 200 µL of each extract was transferred into a clear, flat-bottom 96-well plate. Absorbance was measured at 562 nm using a microplate reader. CPC extraction solution without ARS was used as the reagent blank. Samples with absorbance values exceeding the linear detection range were diluted using the same CPC extraction solution, and the measured values were multiplied by the corresponding dilution factor.

Acellular static-soft, static-stiff, and dynamic hydrogels were subjected to the same fixation, staining, washing, and CPC-extraction procedures to account for condition-dependent nonspecific retention of ARS by the GelMA networks. The absorbance obtained from the corresponding acellular hydrogel control was subtracted from each cell-containing sample.

### 4.14. YAP and F-Actin Immunofluorescence Staining

BMSCs cultured within static-soft, static-stiff, and dynamic soft-to-stiff GelMA hydrogels were fixed with 4% methanol-free formaldehyde in PBS for 15 min at room temperature. Samples were washed at least twice with PBS and permeabilized with 0.1% Triton X-100 in PBS for 15 min at room temperature. After additional PBS washes, nonspecific binding was blocked with 1% bovine serum albumin in PBS for 30–45 min at room temperature. The samples were then incubated with an Alexa Fluor 488-conjugated rabbit monoclonal antibody against YAP at a dilution of 1:500 for 1 h at room temperature in the dark. After three washes with PBS for 5 min each, F-actin was stained with a far-red-conjugated phalloidin reagent at a dilution of 1:400 for 30–60 min at room temperature in the dark. Nuclei were subsequently counterstained with DAPI at a dilution of 1:1000, corresponding to a final concentration of 1 µg/mL, for 5 min at room temperature. Fluorescence images were acquired using an LSM 900 confocal laser-scanning microscope (Carl Zeiss Microscopy GmbH, Jena, Germany) with the DAPI, Alexa Fluor 488, and red channels for nuclei, YAP, and F-actin, respectively.

### 4.15. Quantification of YAP Nuclear Localization

YAP nuclear localization was quantified using ImageJ/Fiji. Nuclear regions of interest were defined from the DAPI channel. Whole-cell boundaries were identified using the F-actin channel, and cytoplasmic regions were generated by subtracting the nuclear region from the corresponding whole-cell region.

The mean YAP fluorescence intensity was measured separately within the nuclear and cytoplasmic regions. Background fluorescence was measured from cell-free regions and subtracted from both nuclear and cytoplasmic measurements. The YAP nuclear-to-cytoplasmic fluorescence intensity ratio was calculated as:

$$\mathrm{YAP nuclear-to-cytoplasmic}\mathrm{ratio}=\frac{I_{\mathrm{nuclear}}}{I_{\mathrm{cytoplasmic}}}$$

where *I*_nuclear_ and *I*_cytoplasmic_ represent the background-corrected mean YAP fluorescence intensities within the nuclear and cytoplasmic regions, respectively.

### 4.16. Statistical Analysis

Quantitative data are presented as mean ± SD from at least three independent hydrogels or experiments. Technical replicates and multiple fields from the same hydrogel were averaged before analysis. Statistical analyses were performed using GraphPad Prism version 8.0.2 (GraphPad Software, San Diego, CA, USA). Comparisons between two groups were performed using a two-tailed unpaired Student’s *t*-test. One-way ANOVA followed by Tukey’s multiple-comparisons test was used for comparisons among three or more groups. Two-factor datasets were analyzed using two-way ANOVA followed by Tukey’s multiple-comparisons test; repeated-measures two-way ANOVA was used when the same samples were measured over time. Statistical significance was defined as *p* < 0.05: ns, not significant; * *p* < 0.05; ** *p* < 0.01; *** *p* < 0.001; and ****p < 0.0001.

## Data Availability

The datasets generated and analyzed during this study can be obtained from the corresponding author upon reasonable request.

## Supplementary Materials

The following supporting information can be downloaded at: https://www.mdpi.com/article/10.3390/gels12100920/s1, Supplementary Materials and Methods; Figure S1: Effects of 405 nm light exposure on cell viability; Figure S2: Expression of macrophage phenotype-associated markers; Figure S3: Verteporfin attenuates dynamic-stiffening-enhanced osteogenic differentiation.
