# Supplementary material for: Temporal Matrix Stiffening Programs Osteogenic Differentiation and Immunomodulation to Enhance Osteogenesis
Source: Gels. 2026 Oct 14;12(10):920. doi: 10.3390/gels12100920 (PMC13704329; doi:10.3390/gels12100920)
Supplement: Supplementary file 1 [file gels-12-00920-s001.zip › gels-4503641-supplementary.pdf]

# SUPPORTING INFORMATION

## Temporal Matrix Stiffening Programs Osteogenic Differentiation and Immunomodulation to Enhance Osteogenesis

Bofeng Pan[1], Jose-Enrique Rodarte[1,2], Jay Ming Tong[1,2], Ethan Nabeta[1], and Dake Hao[1,3,*]

[1] Department of Surgery, University of California Davis, Sacramento, CA 95817, USA

[2] Department of Biomedical Engineering, University of California Davis, Davis, CA 95616, USA

[3] Shriners Children's, Northern California, Sacramento, CA 95817, USA

** Correspondence: dkhao@health.ucdavis.edu*

## Contents

**Supplementary Materials and Methods**

| | |
|---|---|
| **Figure S1** | Effects of 405 nm light exposure on BMSC and macrophage viability |
| **Figure S2** | Expression of macrophage phenotype-associated markers |
| **Figure S3** | Verteporfin attenuates dynamic-stiffening-enhanced osteogenic differentiation |

### Supplementary Materials and Methods

Unless otherwise specified, THP-1 cell differentiation, BMSC culture, osteogenic induction, GelMA preparation, and the generation of static-soft, static-stiff, and dynamic soft-to-stiff hydrogel conditions were performed as described in the main manuscript. Cell-laden hydrogels contained 10% (w/v) GelMA and 0.02% (w/v) lithium phenyl-2,4,6-trimethylbenzoylphosphinate (LAP). The dynamic group was initially prepared using the static-soft photocrosslinking conditions and subsequently stiffened on Day 2 by secondary exposure to 405 nm light at 30 mW/cm$^2$ for 30 s.

#### Assessment of cell viability following 405 nm light exposure

BMSCs and THP-1-derived macrophages were evaluated separately. On Day 2, cells were either exposed to 405 nm light at 30 mW/cm$^2$ for 30 s or maintained as no-light controls. The no-light samples underwent the same handling procedure without illumination. All cells were returned to culture immediately after treatment. 24 hours later, on Day 3, the samples were rinsed with PBS and stained using a Live/Dead viability assay according to the manufacturer's instructions. Fluorescence images were acquired using identical exposure, gain, and illumination settings within each cell type. Live and dead cells were identified from the green and red fluorescence channels, respectively. Cell viability was calculated as:

$$\text{Cell viability (\%)} = \frac{\text{Number of live cells}}{\text{Number of live cells} + \text{Number of dead cells}} \times 100$$

Multiple imaging fields were analyzed for each hydrogel and averaged to generate one biological replicate. Three independent hydrogels were analyzed per condition.

**Immunofluorescence staining of macrophage phenotype-associated markers**

THP-1-derived macrophages cultured within static-soft, static-stiff, or dynamic soft-to-stiff GelMA hydrogels were analyzed on Day 5. Samples were rinsed with PBS, fixed with 4% paraformaldehyde at room temperature, and washed three times with PBS. Because CD86 and CD163 are cell-surface markers, detergent permeabilization was omitted. Nonspecific antibody binding was blocked with 5% bovine serum albumin (BSA) for 1 h at room temperature. The hydrogels were subsequently incubated overnight at 4°C with PE-conjugated mouse anti-human CD86 antibody (R&D Systems, FAB141P; 1:100) and rabbit monoclonal anti-CD163 antibody (Abcam, ab182422; 1:100), diluted in 1% BSA. Approximately 300 μL of antibody solution was added to each sample. The following day, the samples were washed three times with DPBS and incubated with Alexa Fluor 488-conjugated goat anti-rabbit IgG (H+L) secondary antibody (Thermo Fisher Scientific, A-11008; 1:500) for 1 h at room temperature in the dark. After three additional DPBS washes, the samples were mounted using ProLong Diamond Antifade Mountant with DAPI (Thermo Fisher Scientific, P36962). CD86-PE, CD163–Alexa Fluor 488, and DAPI fluorescence were acquired using sequential scanning. Identical laser power, detector gain, exposure, and image-processing parameters were maintained across all experimental groups. Mean fluorescence intensity (MFI) was quantified from background-corrected cell-containing regions of interest using ImageJ/Fiji. Multiple imaging fields were analyzed and averaged to generate one value for each independent hydrogel. Three independent hydrogels were analyzed per condition.

**Verteporfin inhibition experiment**

Macrophage-conditioned media (CM) were generated under static-soft, static-stiff, and dynamic soft-to-stiff GelMA conditions as described in the main manuscript. CM collected on Day 5 was combined with fresh osteogenic medium at a 1:1 ratio and applied to BMSCs cultured in the corresponding hydrogel condition. Verteporfin was dissolved in cell-culture-grade DMSO to prepare a 10 mM stock solution. The stock was diluted to a 0.5 mM intermediate solution and subsequently added to the culture medium at 1:1,000 to obtain a final Verteporfin concentration of 0.5 μM. Verteporfin solutions were prepared and handled under reduced-light conditions. For the dynamic group, secondary photocrosslinking was completed on Day 2 before adding Verteporfin-containing medium, thereby preventing Verteporfin exposure to the 405 nm light used for matrix stiffening. Static-soft and static-stiff groups received the corresponding treatment at the same time. Cultures without Verteporfin were maintained in parallel. Fresh medium containing 0.5 μM Verteporfin was supplied at each scheduled medium change. Alkaline phosphatase (ALP) activity was measured on Day 7 and osteocalcin (OCN) concentration was measured on Day 14 using the same assay procedures and normalization methods. Three independent hydrogels were analyzed per condition.

**Statistical analysis**

Data are presented as mean ± standard deviation, with each independent hydrogel treated as one biological replicate. Cell viability between the no-light and light-exposed groups was compared using an unpaired two-tailed Student's *t*-test. CD86 and CD163 MFI values were analyzed using ordinary one-way ANOVA followed by Tukey's multiple-comparisons test. Verteporfin experiments were analyzed using ordinary two-way ANOVA, with hydrogel condition and Verteporfin treatment as the two factors, followed by Tukey's multiple-comparisons test for pairwise comparisons among the experimental groups. A value of $p < 0.05$ was considered statistically significant.

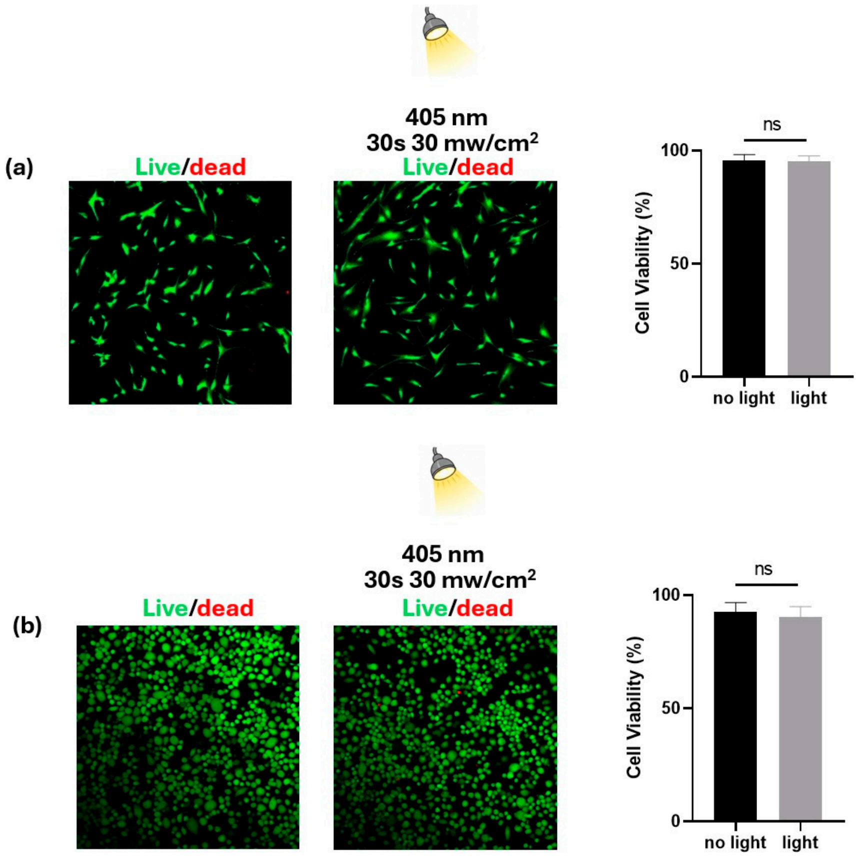


**Figure S1. Effects of 405 nm light exposure on cell viability.** On Day 2, cell-laden hydrogels were either maintained as no-light controls or exposed to 405 nm light at 30 mW/cm² for 30 s. Live/Dead staining was performed 24 h later on Day 3. Representative fluorescence images and corresponding viability quantification are shown for (a) BMSCs and (b) THP-1-derived macrophages. Live and dead cells are displayed in green and red, respectively. Data are presented as mean ± SD (n = 3 independent hydrogels). Statistical significance was determined using an unpaired two-tailed Student's t-test. ns, not significant.

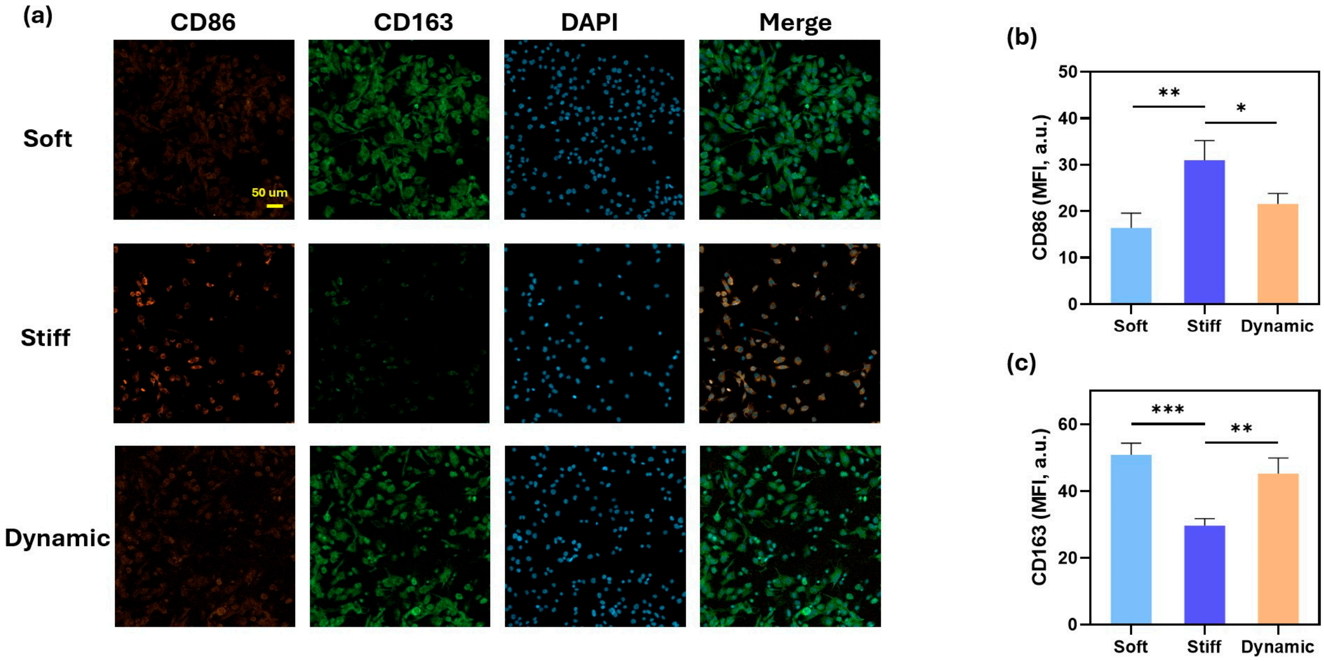


**Figure S2. Expression of macrophage phenotype-associated markers.** (a) Representative immunofluorescence images of the M1-associated marker CD86 (orange), the M2-associated marker CD163 (green), and DAPI-stained nuclei (blue) in THP-1-derived macrophages cultured within static-soft, static-stiff, and dynamic soft-to-stiff GelMA hydrogels on Day 5. Scale bar: 50 μm. (b, c) Quantification of CD86 and CD163 mean fluorescence intensity (MFI), respectively. Data are presented as mean ± SD ($n = 3$ independent hydrogels per condition); measurements from multiple imaging fields were averaged to obtain one value for each hydrogel. Statistical significance was determined using one-way ANOVA followed by Tukey's multiple-comparisons test. *$p < 0.05$, **$p < 0.01$, and ***$p < 0.001$.

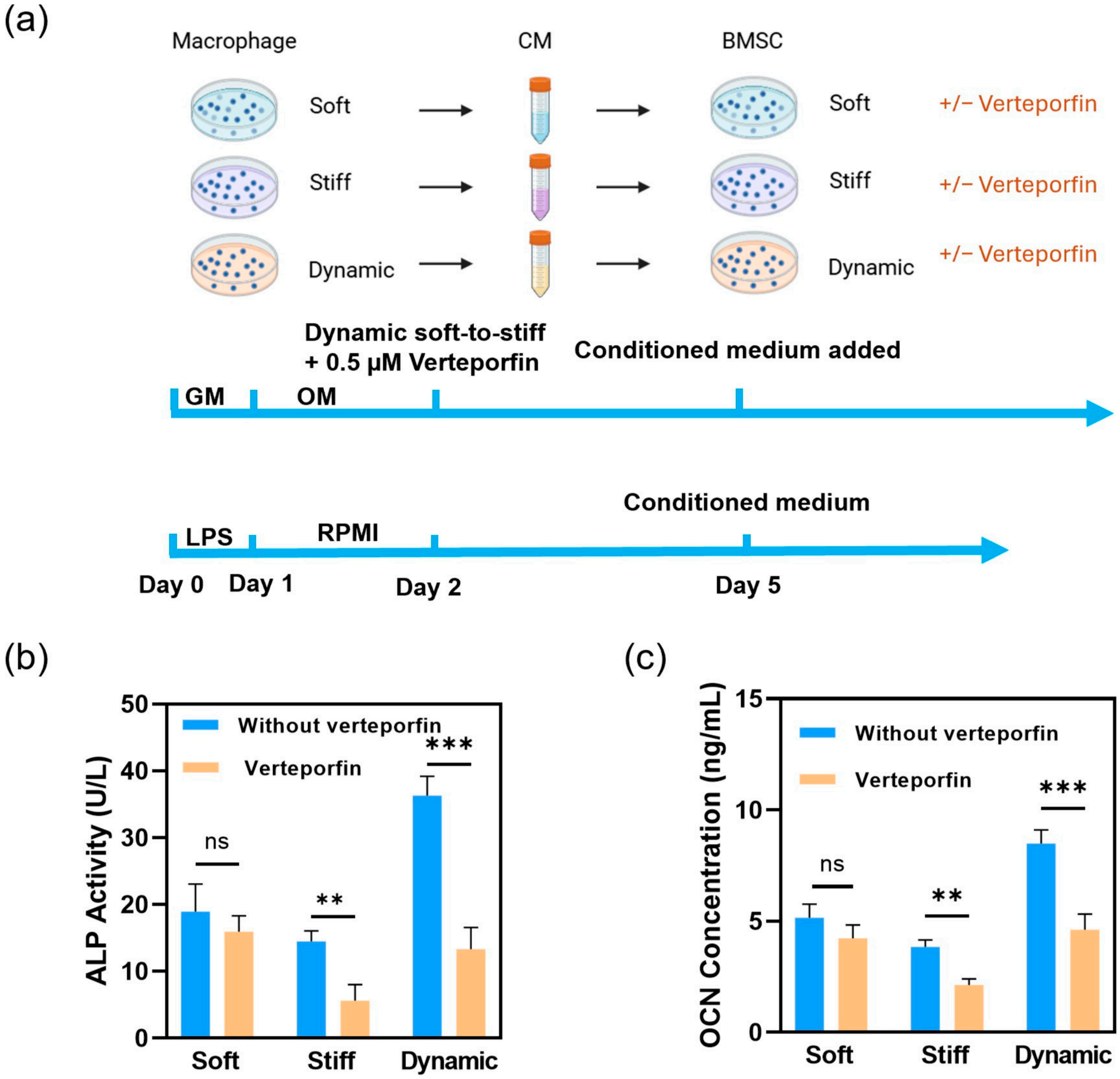


**Figure S3. Verteporfin attenuates dynamic-stiffening-enhanced osteogenic differentiation.** (a) Schematic of the experimental design. Macrophage-conditioned media (CM) generated under static-soft, static-stiff, and dynamic soft-to-stiff hydrogel conditions were applied to BMSCs cultured in the corresponding hydrogels. In the dynamic groups, secondary stiffening was induced on Day 2 using 405 nm light at 30 mW/cm² for 30 s. Immediately after light exposure, BMSC cultures received medium with or without 0.5 μM Verteporfin. (b) ALP activity and (c) OCN concentration under the indicated conditions. Data are presented as mean ± SD (n = 3 independent hydrogels per condition). Statistical significance was determined using ordinary two-way ANOVA with hydrogel condition and Verteporfin treatment as factors, followed by Tukey's multiple-comparisons test. ns, not significant; $^{**}p < 0.01$, and $^{***}p < 0.001$. GM, growth medium; OM, osteogenic medium; CM, conditioned medium.
